# PureCLIP: capturing target-specific protein–RNA interaction footprints from single-nucleotide CLIP-seq data

**DOI:** 10.1186/s13059-017-1364-2

**Published:** 2017-12-28

**Authors:** Sabrina Krakau, Hugues Richard, Annalisa Marsico

**Affiliations:** 1 Max Planck Institute for Molecular Genetics, Ihnestrasse 63–73, Berlin, 14195 Germany; 2 Sorbonne Universités, UPMC Univ Paris 06, CNRS, IBPS, UMR 7238, Laboratoire de Biologie Computationnelle et Quantitative (LCQB), 4 place Jussieu, Paris, 75005 France; 3 Freie Universität Berlin, Takustr. 9, Berlin, 14195 Germany

**Keywords:** Protein–RNA interaction, iCLIP-seq, eCLIP-seq, Crosslink sites, Hidden Markov model

## Abstract

The iCLIP and eCLIP techniques facilitate the detection of protein–RNA interaction sites at high resolution, based on diagnostic events at crosslink sites. However, previous methods do not explicitly model the specifics of iCLIP and eCLIP truncation patterns and possible biases. We developed PureCLIP (https://github.com/skrakau/PureCLIP), a hidden Markov model based approach, which simultaneously performs peak-calling and individual crosslink site detection. It explicitly incorporates a non-specific background signal and, for the first time, non-specific sequence biases. On both simulated and real data, PureCLIP is more accurate in calling crosslink sites than other state-of-the-art methods and has a higher agreement across replicates.

**Electronic supplementary material:**

The online version of this article (doi:10.1186/s13059-017-1364-2) contains supplementary material, which is available to authorized users.

## Background

The interactions between RNAs and RNA binding proteins (RBPs) play essential roles in both transcriptional and post-transcriptional gene regulation. RBPs bind on several sites of both coding and non-coding RNAs with a more or less strong binding affinity for both RNA sequence and structure. To understand fully the regulatory processes mediated by RBPs, it is crucial to determine accurately the full landscape of interactions for a protein of interest. State-of-the-art technologies using crosslinking and immunoprecipitation combined with high-throughput sequencing (CLIP-seq) allow genome-wide binding site detection with high resolution. The most commonly used protocols in this field are HITS-CLIP [1], photoactivatable ribonucleoside-enhanced CLIP (PAR-CLIP) [2] and since 2010, individual-nucleotide CLIP (iCLIP) [3]. All methods use UV light, which causes the formation of crosslinks at protein–RNA interaction sites. These crosslinks subsequently increase the probability for base transitions, deletions, and truncations during the reverse transcription. Such *diagnostic events* can be used to localize the crosslink position. However, due to the ligation of an adapter at the 5^′^ end of the RNA fragments, the HITS-CLIP and PAR-CLIP methods capture only cDNAs that are entirely read by the reverse transcriptase, i.e., not truncated. The fraction of truncated and thus, lost fragments is typically over 80 *%* [4].

iCLIP-seq uses a cleavable adapter in combination with an additional circularization step, which allows all cDNA fragments to be amplified and sequenced. As a consequence, valuable information about the exact crosslink site can be retained from truncated cDNAs, or more precisely from the read start sites they cause. Recently, various improvements to the protocol were proposed to alleviate previous limitations [5, 6]. Another protocol called eCLIP was published in 2016 [7]. Like iCLIP, it provides single-nucleotide resolution by capturing truncated cDNAs but, due to the optimization of several steps, it improves the specificity of called binding sites. To date, eCLIP datasets for more than 120 different proteins have been published by the ENCODE consortium [8, 9]. While previous CLIP-seq experiments often had matched IgG control experiments, which suffer from sparsity and high amplification rates [7], the eCLIP-seq protocol is designed to generate a size-matched input control. This input control is sampled prior to the immunoprecipitation and thus, contains the signal of a non-specific background.

To infer target-specific RBP binding regions from iCLIP/eCLIP data, it is crucial to account for different sources of biases, such as transcript abundances, crosslinking sequence preferences [4], and mappability. The crosslinking sequence bias can also be observed within the eCLIP input data, since it “represent[s] RNAs crosslinked to many different RBPs and should reflect the sequence preferences at crosslink sites that are common to a mixture of RBPs” [5]. Haberman et al. showed that certain polypyrimidine-rich k-mers, which they call crosslink-associated (CL) motifs, are enriched at read start sites in both input and target eCLIP data compared to upstream regions [5].

Besides background noise, such as the signal from sticky RNA fragments or non-specific crosslink events within CL motifs, the binding of background proteins is a major challenge in the analysis of CLIP-seq data. A recent study analyzing previously published PAR-CLIP datasets showed that if no control dataset is used for correction, up to 45 *%* of the called binding sites overlap with background binding sites [10]. Background binding regions that are common to several CLIP-seq datasets have been systematically identified [11] and can be used to validate called binding sites. These findings demonstrate the importance of control experiments, such as input experiments, to reduce the number of false positives at such regions.

Several tools have been developed for the computational analysis of HITS-CLIP and PAR-CLIP data [12–14], but very few tools have been developed that are tailored for the specific analysis of iCLIP/eCLIP data. In addition, previous methods for CLIP-seq data analysis do not fully take into account possible sources of bias, such as transcript abundances and non-specific CL motifs, which heavily affect iCLIP and eCLIP data [5, 15], thereby they return a high number of false calls. The tool Piranha [13] performs strand-specific peak-calling. It supports the incorporation of covariates, but does not explicitly normalize for a non-specific background signal. It models the underlying bin-wise read count distribution to compute a genome-wide significance threshold above which peaks are called. CLIPper [7] is also a strand-specific peak-calling method designed by members of the ENCODE Consortium to analyze published eCLIP datasets. It incorporates annotations from the reference genome and computes significance thresholds gene by gene. Both tools, Piranha and CLIPper, are peak-calling methods that do not detect individual crosslink sites. Their limitation is that they potentially miss low-affinity binding regions with a clear iCLIP truncation pattern due to the arbitrary setting of a threshold on the number of reads. In addition, they are sensitive to call peaks, which is caused, for example, by artifacts within high abundant RNAs. The CITS method on the other hand aims to call individual crosslink sites from iCLIP-seq data [16]. It clusters reads based on their start sites and uses a statistical test to detect sites within such clusters containing a significant fraction of read starts. A drawback of this method is that it does not explicitly model the relation between read start counts and the read coverage generated by pulled-down iCLIP fragments. As a result, it might also be sensitive to artifacts within highly abundant RNAs. In contrast, PIPE-CLIP [17] is an online pipeline for the analysis of HITS-CLIP, PAR-CLIP, and iCLIP data designed to call peaks and crosslink sites separately, which are subsequently merged. Although constituting a powerful idea, one drawback of this method is that it is not designed to include control experiments in the analysis. In addition, being designed to be an online method, its application for transcriptome-wide analysis is not practically feasible. As described above, both CLIP-seq peak-calling methods and individual crosslink site detection methods have advantages and disadvantages, but currently no method exists that addresses peak-calling and individual crosslink site detection simultaneously while correcting for possible biases.

We have developed PureCLIP, a method to capture target-specific protein–RNA interaction footprints from iCLIP/eCLIP-seq data. PureCLIP calls individual crosslink sites considering both regions enriched in protein-bound fragments and iCLIP/eCLIP specific truncation patterns. Our method uses a non-homogeneous hidden Markov model (HMM) to incorporate additional factors into the model, such as a non-specific background signal from input experiments and CL motifs, to reduce the number of false positives. We have exhaustively validated the superiority of PureCLIP over several existing methods in various settings. First, we designed a realistic iCLIP/eCLIP simulation setup and demonstrated that, over a wide range of simulation parameters, PureCLIP is up to 7–15 % more precise than other methods in detecting target-specific crosslink sites. Second, due to the lack of an experimental gold standard, we selected four datasets of published iCLIP/eCLIP data for evaluation where the RBP motif or the predominant binding region of the RBP is known. We consistently observed that PureCLIP is better than other methods in determining bona fide binding site locations. In particular, the incorporation of covariates, such as the input signal and CL motifs, increases the precision of PureCLIP up to 8–10 % compared to previous methods. Third, the replicate agreement of target-specific crosslink sites called by PureCLIP is up to 8–20 % higher than other methods, indicating that PureCLIP is highly specific in crosslink site detection.

## Results

### Overview of the approach

**Fig. 1:**
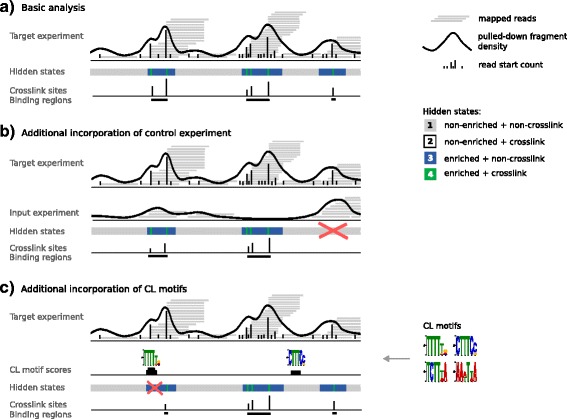
Overview of the PureCLIP approach. **a** PureCLIP starts with mapped reads from a target iCLIP/eCLIP experiment and derives two signals: the pulled-down fragment density and individual read start counts. Based on these two observed signals, it infers for each position the most likely hidden state. The goal is to identify all sites with an *enriched* + *crosslinked* state. Individual crosslink sites can then be merged to binding regions. **b** Additionally, information from input control experiments can be incorporated. Its fragment density is used to correct for a non-specific background signal, which reduces the number of false calls. **c** Furthermore, PureCLIP can incorporate information about CL motifs to reduce false calls caused by non-specific crosslinks. CL crosslink-associated

PureCLIP aims to detect individual crosslink sites originating from interactions between RNAs and the protein targeted by the experiment. To accomplish this, we address two objectives: (1) detecting regions enriched in mapped reads caused by pulled-down RNA fragments and (2) detecting crosslink sites where a significant fraction of read starts accumulate at the same position, originating from truncated cDNAs (Fig. 1a).

In the following, we give an overview of how we derive this information, assuming that the given data are iCLIP/eCLIP-seq reads that have been mapped to either a genome or a transcriptome and that polymerase chain reaction (PCR) duplicates have been removed. The output of PureCLIP consists of individual crosslink sites associated with a score. Since multiple crosslink sites can occur within one binding region, the crosslink sites are optionally merged.

#### Hidden Markov model

CLIP-seq data feature a spatial dependency between neighboring positions. Inferring crosslink sites from the observed data can be considered to be a segmentation problem and we address this using a HMM. The HMM has a single-nucleotide resolution and each position can be categorized either as *non-enriched* or *enriched*, indicating whether the position is enriched or not in protein bound fragments. In addition, each position can also be categorized as *non-crosslink* or *crosslink*, indicating whether it represents a crosslink site or not. This combination results in four hidden states: (1) *non-enriched* + *non-crosslink*, (2) *non-enriched* + *crosslink*, (3) *enriched* + *non-crosslink*, and (4) *enriched* + *crosslink* (Fig. 1). State (2) corresponds to non-specific crosslink sites and it is included in the model for mathematical completeness. We are interested in all sites with a hidden state (4), i.e., sites that are enriched in pulled-down RNA fragments and show the truncation pattern (Fig. 1a).

To detect *enriched* + *crosslinked* sites, PureCLIP uses two signals derived from the mapped reads: (1) the *pulled-down fragment density*, which is a smoothed signal derived from the read start counts and holds information about the enrichment within the current region, and (2) the read start counts themselves, which hold information about potential truncation events (Fig. 1). Importantly, for the first signal (1), we do not use position-wise read counts, since for iCLIP/eCLIP data these are strongly influenced by truncation events in the neighborhood. On the other hand, using counts within larger bins would not be very accurate in estimating the position-wise signal of the pulled-down fragments. To address this problem, we compute a Gaussian kernel density estimate [18] for each position based on the raw read start counts. Then one type of distribution is used to model these pulled-down fragment densities, with one set of parameters for the *non-enriched* state and one for the *enriched* state, assuming that the *enriched* state is more likely to cause high fragment density values than the *non-enriched* state. Similarly, read start counts are modeled under the assumption that the *crosslink* state is more likely to generate a higher fraction of reads starting at one position than the *non-crosslink* state. To account for differently covered regions, the parameters of the read start count distributions at individual positions depend on the pulled-down fragment density.

The fragment density distributions and the read start count distributions are combined to obtain the emission probabilities of each of the four hidden states. For each position, we can then address the question: which of the four hidden states most likely caused the observed data?

#### Incorporation of additional factors into the PureCLIP model

The observed signals can be biased by a number of different factors, such as transcript abundance or crosslinking sequence preferences. An important feature of PureCLIP is the incorporation of position-wise external data into the HMM framework to correct for such biases. We do this using generalized linear models, while distinguishing between different types of covariates.

We expect regions within highly abundant RNAs to show more read start counts than regions within less abundant RNAs. This holds for both target binding regions and for regions with non-specific background noise. To normalize for this, information from input control experiments can be included to influence the emission probability distributions of the *non-enriched* and *enriched* states. With this, we aim to reduce the number of false positives for highly abundant RNAs (see Fig. 1b) while increasing the sensitivity for less abundant RNAs.

Furthermore, we expect a higher number of read start counts, for example, at positions within CL motifs. Thus, to correct for the crosslinking sequence bias, information about CL motifs can be incorporated (see Fig. 1c) to influence the *non-crosslink* and *crosslink* emission distributions.

### Evaluation of the performance of PureCLIP in comparison to previous strategies

Evaluating a method’s performance in analyzing CLIP-seq data is not trivial, since no gold standard of binding regions or crosslink sites exists. To address this: (1) We assess the precision and recall of PureCLIP in basic mode, i.e., without additional covariates, in calling individual crosslink sites on simulated data. (2) We then use real iCLIP and eCLIP datasets of proteins with known binding characteristics, such as known sequence motifs or known predominant binding regions. We assess the ratio of sites called by each method that fall within these motifs or inside those binding regions. Called crosslink sites within such regions are defined as true positives. Here we applied PureCLIP in four different settings: in basic mode, incorporating the input signal, incorporating CL motifs, and incorporating both the input signal and CL motifs simultaneously. Although extremely valuable, this evaluation approach is limited since it is unknown how far the protein of interest can also bind to alternative motifs or outside the defined bona fide binding regions. For this reason, (3) we also assessed the agreement of called crosslink sites between eCLIP replicates.

We compared PureCLIP with a range of previous strategies, most importantly CITS [16], which, like PureCLIP, can call individual crosslink sites rather than broader peak regions. Additionally, since to date no other tool exists that addresses both peak-calling and crosslink site detection simultaneously for truncation-based CLIP-seq data, we combine the peak-calling methods Piranha [13] and CLIPper [19] with CITS. More precisely, we use the intersection of the called peaks and the CITS crosslink sites. While this intersection depends on the selected *p* value thresholds for both methods, the resulting sites are scored in two different ways, using either the score from the peak-calling method (referred to as Piranha ^sc^ or CLIPper ^sc^) or from CITS (referred to as CITS ^sc^) (for details see “Methods”). With this, we aim to cover the range of currently available strategies for detecting protein–RNA interactions at single-nucleotide resolution. To ensure a comparative assessment that is as impartial as possible, we also compared PureCLIP with combinations based on different *p* value thresholds and found that these do not affect the results (see Additional file 1: Figure S14).

Additionally, we applied the simplest possible approach, namely calling all sites with a read start count above a certain threshold. This gives us an understanding of how different methods perform in different scenarios compared to this naive approach. In the following, we refer to this as the *simple threshold* method.

### PureCLIP outperforms previous strategies on simulated iCLIP/eCLIP-seq data

Since the only available CLIP-seq simulator [20] is limited to PAR-CLIP and HITS-CLIP data, we implemented our own simulation workflow to mimic the experimental steps of the iCLIP and eCLIP protocols. Starting from real RNA-seq data and known binding regions of a certain protein, our simulation aims to reproduce the characteristics of iCLIP/eCLIP data as accurately as possible. To simulate a target signal, our workflow uses aligned RNA-seq data. It pulls down a certain fraction of the fragments that cover a known binding region and then applies truncations according to a given rate (for details, see “Methods”). Furthermore, the non-specific binding of background proteins is simulated using published common background regions and random noise from RNA-seq data is added.

**Fig. 2:**
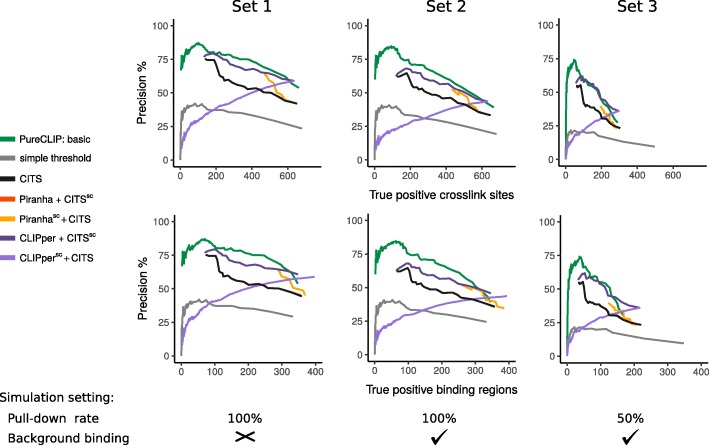
Performance on simulated iCLIP-seq data. Precision vs number of true positive crosslink sites (top) and vs number of true positive binding regions, i.e., regions with at least one correctly called crosslink site (bottom) for three different simulation settings. The characteristics of each simulation are reported below the plots. All strategies were applied using different sensitivity settings, i.e., different *p* value or score thresholds. The leftmost point of each curve corresponds to the number of true positives associated with the lowest *p* value or the highest score the strategy can report. The curves of Piranha + CITS ^sc^ overlap with those of CITS

To evaluate the performance of PureCLIP under different conditions, we produced three different datasets. For these, we used varying pull-down rates for the target signal, i.e., either 100 or 50 % of the RNA fragments that overlap a target binding region are selected and further modified where required. Reducing the pull-down rate enables us to get an idea for how the different methods perform for proteins with overall lower binding affinities. Additionally, we simulated non-specific background binding for two of the datasets (see Fig. 2).

For the evaluation, we define a called crosslink site as a true positive if a target crosslink site was simulated at the same position. The precision of a method is calculated as the fraction of true positives among the called crosslink sites. We first investigated the precision versus the number of true positive crosslink sites. The results in Fig. 2 (top) demonstrate that PureCLIP reaches a higher precision in detecting individual crosslink sites than previous strategies for all simulation settings. In particular for the top-ranking sites, it has a far better precision compared to other methods, while being comparable to CITS ^sc^ + CLIPper for more sensitive settings. However, it is worth mentioning here that sensitive settings that are characterized by a precision below 50 % are generally not of interest.

Furthermore, we investigated whether the crosslink sites called by PureCLIP could be used to recover target binding regions (i.e., known binding regions in which crosslink sites were simulated) or if they cluster within a few regions with high fragment density. A target binding region is counted as a true positive, if it could be recovered with at least one called crosslink site. The precision is defined as the percentage of called crosslink sites within target binding regions. For all simulation settings, the results show that PureCLIP recovers binding regions with higher precision compared to previous strategies (Fig. 2, bottom).

### PureCLIP detects bona fide binding regions with higher precision compared to previous strategies

We used publicly available eCLIP (PUM2, RBFOX2, and U2AF2) [7] and iCLIP (U2AF2) [21] datasets to measure the performance of the different strategies in calling crosslink sites within bona fide binding regions. For PUM2 and RBFOX2, these binding regions were defined by their known sequence motifs (see Additional file 1: Figure S1), while for U2AF2, we make use of its known predominant binding region ∼11 nt upstream of 3^′^ splice sites [21]. Here, a sequence motif based definition of the binding region is not applicable, since U2AF2 binds to poly(U) tracts, which coincide with non-specific CL motifs.

**Fig. 3:**
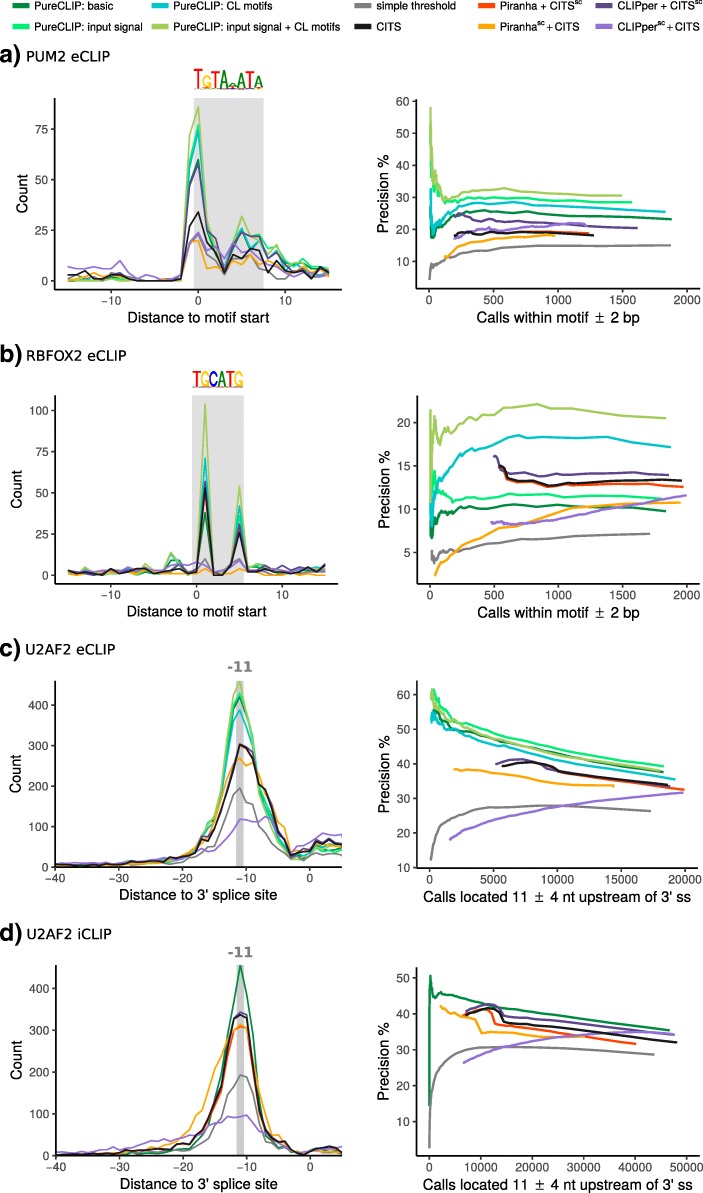
Accuracy in detecting bona fide binding regions (depicted by gray areas). Left panel: **a** Distribution of the distances of the top 1000 sites called by each method to the closest PUM2 motif start position. **b** Same as (**a**), but for RBFOX2 motif start positions. **c**, **d** Distribution of the distances of the top 5000 sites called by each method with respect to 3^′^ splice sites. Right panel: Precision of the called sites for all methods at different sensitivity settings, i.e., using different *p* value or score thresholds. The leftmost point of each curve corresponds to the number of calls within the bona fide binding region associated with the lowest *p* value or highest score the strategy can report

For the PUM2 data, all strategies revealed an accumulation of called crosslink sites at the 5^′^ end of PUM2 motif occurrences and another slightly weaker accumulation towards the 3^′^ end (Fig. 3a, left panel). For RBFOX2 eCLIP data, we observe an accumulation of called crosslinks at the two guanines within the motif (Fig. 3b, left panel). These crosslinking patterns are in agreement with previous studies [7, 16] and, since crosslinks do not preferentially occur at guanines, are most likely caused by target-specific protein–RNA interactions.

#### Performance of PureCLIP without incorporating external data as covariates

We first investigated the precision of PureCLIP in basic mode, i.e., without the incorporation of any covariates, where calls are considered true positives if they fall within the motif area or upstream of 3^′^ splice sites. We observed that PureCLIP outperforms all other methods even without covariates in three out of four datasets, as shown in Fig. 3 (right panel). Interestingly, when applying strategies that merge results from peak-calling tools and CITS using the peak-calling scores for ranking (Piranha ^sc^ + CITS and CLIPper ^sc^ + CITS), we always get a lower precision than when using the CITS crosslink site detection score for ranking (Piranha + CITS ^sc^ and CLIPper + CITS ^sc^).

#### Incorporation of input control data improves crosslink site detection

We expect the observed pulled-down fragment densities to be biased by different factors, among others by RNA transcript abundances. The published eCLIP datasets have input control experiments [7], which provide information about the non-specific background signal, i.e., RNA fragments crosslinked to background proteins. We observe significant correlations between the fragment density of the eCLIP target dataset and the input dataset with Pearson correlation coefficients ranging from 0.36 to 0.42 (*p* values <2.2×10^−16^) (see Additional file 1: Figure S12a). Therefore, the incorporation of the input signal into the PureCLIP framework gives us the possibility to indirectly normalize for transcript abundances, crosslinking preferences, and other local biases.

In detail, the PureCLIP framework uses the eCLIP input signal to model the emission probabilities of the *non-enriched* and *enriched* states for the observed data, i.e., the pulled-down fragment densities. This means that instead of using one global emission probability distribution for the *non-enriched* or *enriched* states, the position-wise input signal is used to model the expected mean parameter of each of the two emission probability distributions (see Additional file 1: Figure S12b). With this, we aim to reduce the number of false positives, for example, within highly abundant RNAs while increasing the sensitivity within lowly abundant RNAs. The evaluation based on bona fide binding regions from real data shows that incorporating the input signal improves the precision of PureCLIP for all eCLIP datasets and over all sensitivity thresholds (Fig. 3a–c, right panel). In particular, for the top-ranking sites, this greatly improves the precision by reducing the number of false positives in regions with a high non-specific background signal.

#### Incorporation of CL motifs greatly improves crosslink site detection

Another major bias within CLIP-seq data is caused by crosslinking sequence preferences, which also give rise to non-specific crosslink events at sites with no direct interaction between the target protein and the RNA. Hence, this bias influences the individual read start counts. Since our method is designed to detect crosslinking patterns, it also detects a certain fraction of non-target crosslink sites. For PUM2 and RBFOX2, both having known sequence binding motifs distinct from reported CL motifs [5], we observed that 33 and 37 % of the top 1000 sites called by the basic version of PureCLIP overlap with regions harboring a CL motif.

**Fig. 4:**
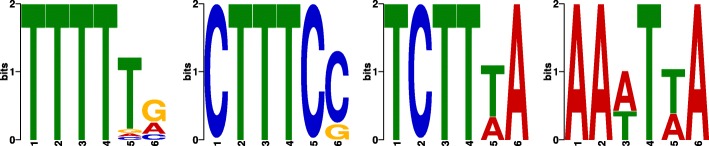
CL motif analysis of PUM2 eCLIP input data. Logo representation of the four top scoring motifs among the first 5000 PureCLIP crosslink sites called on the input dataset. Motifs were detected with DREME and a 10-bp window around the crosslink sites. As previously reported [5], polypyrimidine-rich motifs are overrepresented. CL motifs of the other datasets are shown in Additional file 1: Figure S13

To reduce the number of such potential false positives, we incorporate information about CL motifs into our model. This can be particularly helpful in filtering out non-specific crosslink sites when the protein of interest preferentially binds sequences that are clearly distinct from CL motifs. For this purpose, CL motifs have to be learned first and we do this directly from the data: (1) we call crosslink sites in the eCLIP input data, (2) we then learn CL motifs on these sites using DREME [22], and (3) we apply FIMO [23] to compute the occurrences of those motifs and their scores within the reference genome or transcriptome. These position-wise scores are then incorporated into the HMM framework of PureCLIP to model the emission probabilities of the *non-crosslink* and *crosslink* state for the observed data, i.e., the read start counts. This enables a correction for the crosslinking sequence bias at CL motif positions. As an example, the four most enriched CL motifs from the analysis of PUM2 eCLIP input data are shown in Fig. 4.

The results demonstrate that for PUM2 data (Fig. 3a) and, in particular, for RBFOX2 eCLIP data (Fig. 3b), the incorporation of CL motif scores greatly improves the precision in calling crosslink sites within bona fide binding regions. Interestingly, the simultaneous incorporation of the input signal and CL motif scores improves the precision of PureCLIP even further (Fig. 3a, b). Moreover, we can see that for the protein U2AF2, whose sequence motif coincides with CL motifs, the performance of PureCLIP stays robust and is not impaired by the incorporation of CL motif scores. Altogether, we could see that when incorporating CL motifs, PureCLIP consistently performs better than previous strategies in positioning called sites either at the known binding motif or ∼11 nt upstream of 3^′^ splice sites for U2AF2 (Fig. 3a–d).

### Robustness of PureCLIP over a range of different bandwidths

PureCLIP depends on the bandwidth used for the smoothing of the read start counts when estimating the pulled-down fragment density. The optimal bandwidth depends on the coverage and the given cDNA length distribution, e.g., the longer the cDNAs, the larger the optimal bandwidth. For the evaluations in this study, we used a bandwidth of 50 bp. The results shown in Additional file 1: Figures S15 and S16 demonstrate that PureCLIP reaches a higher precision robustly for a range of different bandwidth parameters, compared to previous strategies.

### PureCLIP has a higher agreement of called sites between eCLIP replicates compared to previous strategies

Besides using known binding regions for evaluation, we aimed to assess the performance of the different methods independently of that information, since in the end the exact binding regions and crosslink sites remain unknown. For this reason, we explored each method’s precision based on the agreement of called crosslink sites between eCLIP replicates, assuming that target-specific binding events are more likely to be observed in both replicates than non-specific noise. We applied all methods to the individual eCLIP replicate datasets and measured for each sensitivity threshold how many of the *x* called crosslink sites in replicate 1 overlap with the top *x* ranking crosslink sites in replicate 2.

Besides target-specific binding events, other factors such as background proteins binding to highly abundant RNAs or the crosslinking sequence bias (see Additional file 1: Section 7) contribute to the measured replicate agreement. We, therefore, count only those sites to the agreement that are also enriched over the input and located outside of regions that are known to be prone to background binding (as published in [11]). Thus, we avoid overestimating the precision of methods that consistently call false crosslink sites in both replicates due to systematic reproducible biases. This potentially also excludes a certain number of true positives that cannot be distinguished from non-specific background noise, but we expect this to affect all methods more or less equally and thus, still allow for a fair comparison. We refer to this measurement as the bias-corrected replicate agreement (see “Methods” for details).

To further prevent a contribution from common non-specific crosslinks, for PUM2 and RBFOX2 we counted only sites to the bias-corrected agreement that are not located within CL motif occurrences. Since the target motifs of these two proteins are clearly distinct from CL motifs, we expect that we do not miss relevant target-specific sites by this. The U2AF2 iCLIP data are excluded from this evaluation, since no input control experiment is available and thus, the bias-corrected replicate agreement cannot be computed.

**Fig. 5:**
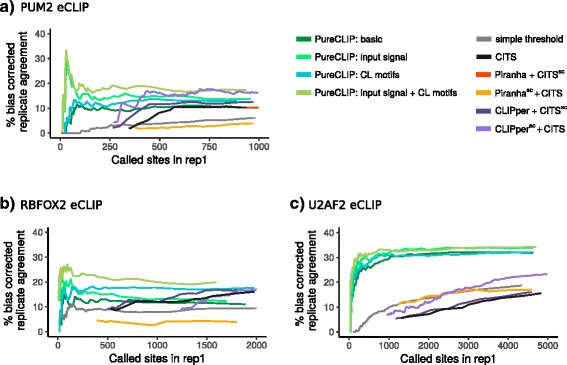
Agreement of called sites between replicates. For each eCLIP dataset (**a**: PUM2, **b**: RBFOX2, **c**: U2AF2), we report for each given number of called sites *x* in replicate 1 (corresponding to a certain *p* value or score threshold), the percentage that were also called within the top *x* ranking sites in replicate 2 after correcting for different biases (see “Results” and “Methods”). The leftmost point of each curve corresponds to the number of calls associated with the lowest *p* value or highest score the strategy can report. rep1 replicate 1

Our evaluations show that PureCLIP has a higher bias-corrected replicate agreement for the top-ranking sites compared to previous strategies, in all four PureCLIP settings and over all three eCLIP datasets, as depicted in Fig. 5. Furthermore, the performance of PureCLIP in basic mode is at least comparable to the other methods, while PureCLIP incorporating the input signal and CL motifs strictly outperforms all other methods. While the individual use of these covariates already improves the agreement, the best results are obtained when both of them are incorporated simultaneously.

Notably, the other strategies show a particularly low bias-corrected replicate agreement within their top-ranking sites. For strategies based on peak-calling scores, this might be due to peaks corresponding to background binding regions. However, except for the *simple threshold* method, the top-ranking sites of all other strategies show a lower agreement before this bias correction in comparison to our method (see Additional file 1: Figures S17a, S18a and S19a).

### PureCLIP captures strongest interaction footprints, not top-ranking peaks

All previous crosslink site detection strategies, and in particular those based on peak-calling scores such as Piranha ^sc^ + CITS and CLIPper ^sc^ + CITS, call more sites in regions of high fragment density than PureCLIP in both basic mode and with the addition of covariates (see Additional file 1: Figures S17f and S18f). Further, the results show that these strategies also call far more sites within known common background binding regions than PureCLIP, even when not incorporating covariates. Moreover, the other strategies have far less bias-corrected agreeing calls between the two eCLIP replicates (Fig. 5). This indicates that the sites within the highest peaks do not necessarily correspond to reproducible target-specific crosslink sites. These findings are in line with the results of the evaluation based on bona fide binding regions (Fig. 3), where strategies based on peak-calling scores (Piranha ^sc^ + CITS and CLIPper ^sc^ + CITS) perform worse than corresponding strategies based on crosslink site detection scores (Piranha + CITS ^sc^ and CLIPper + CITS ^sc^). In other words, most of the CITS sites within top-ranking peaks are not located within regions matching the known binding characteristics of the proteins, and are, thus, likely to be false positives.

### PureCLIP crosslink sites allow accurate detection of larger binding regions

As most of the currently available strategies are designed to call peaks rather than individual crosslink sites, we investigated the performance of PureCLIP at the level of called binding regions for the proteins PUM2 and RBFOX2 using their known sequence motifs. The underlying assumption here is that for called regions, a higher motif density corresponds to a higher accuracy. Therefore, we computed region-wise motif scores, as described in detail in Additional file 1: Section 8. PureCLIP computes binding regions by merging crosslink sites within a certain distance, and here we use the default parameter of 8 bp. Additional file 1: Figure S20 shows that for PUM2, PureCLIP calls regions with a strictly higher accuracy compared to the other evaluated peak-calling methods. For RBFOX2, PureCLIP has a clearly higher accuracy when incorporating CL motifs, while showing a comparable accuracy to other methods when run in its basic mode or only incorporating the input signal. The results show that PureCLIP can detect not only individual crosslink sites but also binding regions around the target motifs with a higher accuracy compared to the evaluated peak-calling methods.

## Discussion

The detection of target-specific protein–RNA interaction sites from single-nucleotide resolution CLIP-seq data is a remaining challenge. Previous methods for the analysis of such data typically suffer from having a large fraction of false positives, as they are sensitive to different sources of biases. Peak callers such as Piranha, which call regions enriched in read coverage without explicitly modeling read start counts at truncation sites, are prone to capturing a high background signal that does not originate from target-specific crosslink events. On the other hand, CITS calls sites with a significant fraction of read starts, but it cannot distinguish whether such sites are caused by target-specific crosslinks or by non-specific crosslinks within highly abundant regions. In addition, CITS does not account for biases, such as different transcript abundances or the crosslinking sequence bias, which can increase the number of false positives.

To overcome these limitations, we propose a new statistical approach called PureCLIP. PureCLIP calls crosslink sites considering both regions enriched in protein-bound fragments and the specifics of iCLIP/eCLIP truncation patterns. It also explicitly models possible sources of bias, such as a non-specific background signal and crosslinking sequence bias to reduce the number of false positives. Both these features, and in particular the incorporation of CL motifs, which represent the non-specific crosslink sequence bias, are an innovation in comparison to existing methods.

A comprehensive evaluation based on simulated and real data has shown that in basic mode, PureCLIP reaches a higher precision compared to previous strategies in almost all cases. Moreover, for real data, the incorporation of input signals and CL motifs additionally improves the precision of PureCLIP in capturing crosslink sites within bona fide binding regions. For the analysis of PUM2 and RBFOX2 data, note that for the 50 top-ranking sites (Fig. 3a, b, right panel), the precision of PureCLIP including the input signal is much higher in comparison to PureCLIP in basic mode or to previous strategies. The results indicate that the top-ranking sites called by other strategies are likely to be caused by a non-specific background signal, which is resolved by PureCLIP when incorporating the input signal.

PureCLIP incorporating CL motif scores strictly outperforms all other strategies over all four datasets. In fact, PureCLIP’s precision in this setting increases, especially for the eCLIP datasets of proteins whose sequence motifs do not coincide with CL motifs, namely PUM2 and RBFOX2 (Fig. 3a, b). For RBFOX2 eCLIP data, the increase is particularly remarkable. This is also the only dataset where PureCLIP without incorporating CL motifs shows a lower precision than strategies that make use of the CITS crosslink site detection score (Fig. 3b). The main reason is that in basic mode, PureCLIP is more sensitive than CITS in also calling non-specific crosslink sites, in particular for this dataset (see Additional file 1: Figure S18d). In general, a high sensitivity is desired, since we also want to detect crosslink sites for low-coverage regions, for example within lncRNAs or for proteins with lower binding affinity. In addition, the number of false positives can be reduced by incorporating CL motifs. Interestingly, when incorporating both the input signal and CL motifs simultaneously, the precision of PureCLIP increases even further, highlighting the huge benefit of the incorporation of both covariates into the model.

Compared to previous strategies, PureCLIP achieves higher agreement in calling RBP-bound sites between eCLIP replicates for bona fide crosslink sites. These are sites where the fragment coverage is enriched over the input signal and they do not overlap known background binding regions and, for PUM2 and RBFOX2, they are not located within CL motif occurrences. Interestingly, the *simple threshold* method, which detects crosslink sites by applying a cutoff on the read start counts, has the worst performance of all on both simulated and real data, as expected, but by far the highest replicate agreement for all datasets when not explicitly accounting for biases. This indicates that, beside target-specific crosslink sites, other factors also contribute to this raw replicate agreement, and that to obtain a meaningful evaluation of all methods, we need to compute a bias-corrected replicate agreement. These results also strongly suggest that it would be valuable in the analysis of iCLIP/eCLIP data to include replicate information explicitly (as already suggested by [15]) but, importantly, this needs to be done carefully while addressing possible sources of biases.

It is also important to stress that for all analyzed eCLIP datasets, PureCLIP calls far fewer crosslink sites within regions of high fragment density (see Additional file 1: Figures S17f, S18f, and S19f) and within known common background binding regions [11] (see Additional file 1: Figures S17e, S18e, and S19e) compared to all other strategies. This even holds for PureCLIP in basic mode. Taken together with PureCLIP’s general higher precision, these findings demonstrate how important it is in the analysis of CLIP-seq data not only to call peaks but also to model accurately the counts of individual read starts, which indicate potential truncation events. This unique feature of PureCLIP enables a distinction to be made between target-specific interaction footprints and non-specific crosslink patterns within highly abundant background binding regions.

Although the main objective of PureCLIP is to detect individual target-specific crosslink sites, it is sometimes desirable to identify larger binding regions for the protein under study. In the current version, PureCLIP can merge crosslink sites into larger binding regions based on their genomic distance. Further work is needed to address this task in a more sophisticated manner. However, the results for simulated data demonstrate that individual crosslink sites also recover a large number of simulated binding regions with higher precision compared to the other strategies. Additionally, the results show that PureCLIP can detect not only individual crosslink sites but also binding regions around the target motifs of PUM2 and RBFOX2 with higher accuracy compared to the evaluated peak-calling methods.

Currently, PureCLIP allows us to incorporate covariates that influence either the emission probabilities of the pulled-down fragment density or the read start counts. Besides information on common background binding regions and replicate agreement, mappability information is a promising candidate that will be investigated further inside the PureCLIP model. Furthermore, the PureCLIP framework could be extended to model non-homogeneous transition probabilities between states, for example, if we want to include information about the sequence or structure binding preferences of the target protein. Given the specifics of the PureCLIP model, besides iCLIP and eCLIP data, it can be used to analyze data from similar single-nucleotide resolution protocols, such as irCLIP [24], iCLAP (cross-linking and affinity purification) [25], and miCLIP (methylation iCLIP), a customized version of iCLIP for capturing m5C methylated sites on RNAs with nucleotide resolution [26]. Additionally, PureCLIP could be adapted to model other types of diagnostic events, such as mutations and deletions, from the PAR-CLIP [2], HITS-CLIP [1], and CRAC [27] protocols.

## Conclusion

More and more high-resolution CLIP-seq datasets are being generated, but the precise determination of protein–RNA interaction sites from iCLIP/eCLIP has been challenging so far. Extensive evaluations demonstrated the superiority of PureCLIP over several previous strategies in detecting target-specific crosslink sites, for both simulated data as well as real datasets. PureCLIP is able to capture protein–RNA interaction footprints precisely, while not relying on the highest peaks, and it is able to correct for biases, such as transcript abundances, background binding, and crosslinking sequence preferences. It, therefore, provides a promising method for analyzing these datasets, and also for proteins with lower binding affinities or proteins binding to low abundant RNAs, such as lncRNAs.

## Methods

### Preprocessing of iCLIP/eCLIP datasets

We analyzed three published eCLIP datasets targeting the proteins PUM2, RBFOX2, and U2AF2 and one iCLIP dataset targeting U2AF2 (see Additional file 1: Table S1 for details).

First, any possible adapter contamination at the 3^′^ ends was removed using TrimGalore on the iCLIP dataset [28] (v0.4.2, based on cutadapt), and by running cutadapt twice on the eCLIP datasets [29] (v1.12). The latter was done to correct for possible double ligation events [7]. Reads shorter than 18 bp were discarded. Next, the reads were mapped against the human genome (hg19) using STAR [30] (v2.5.1b), a read aligner designed for RNA-seq data with setting –alignEndsType EndToEnd, –scoreDelOpen -1 for gap penalty, and –outFilterMultimapNmax 1 to discard reads mapping to multiple locations.

PCR duplicates were removed based on the read mapping positions and the random bar-code sequences (also called UMIs). This is important, as PCR amplification rates are high, in particular for iCLIP datasets. To address this, we used UMI-tools [31], a network based de-duplicating method (with setting –paired), which is able to handle errors within bar-code sequences.

All evaluated datasets come as two replicates. When assessing each method’s ability to recover bona fide binding regions, we pooled the reads of the two replicates, whereas they were analyzed separately when evaluating the agreement between called sites. Due to the differences in the two library preparation protocols [3, 7], we used either the 5^′^-end read (iCLIP) or the 3^′^-end read (eCLIP) of the sequenced fragment for the analysis.

### iCLIP/eCLIP-seq data simulation

**Fig. 6:**
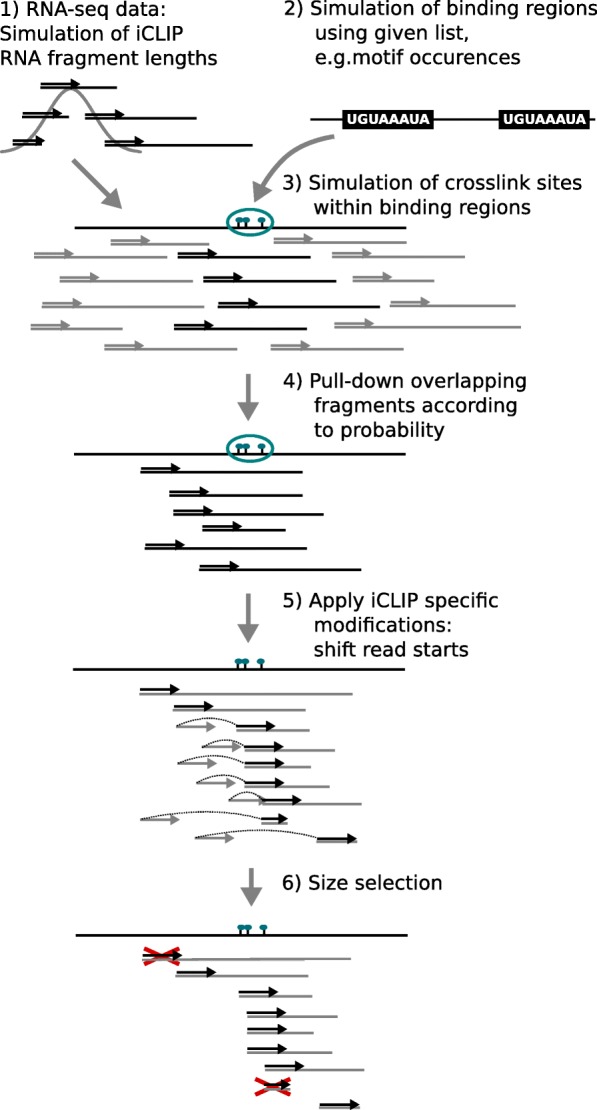
Simulation of iCLIP/eCLIP data
- Binding regions: We use genome-wide PUM2 motif occurrences computed with FIMO [23] to obtain a realistic distribution of binding regions (for details, see Additional file 1: Section 2).
- Crosslink sites: Within each binding region *i*, *c*_*i*_ crosslink sites are drawn uniformly (*c*_*i*_∈{1,…,4}).
- Pull-down: RNA fragments overlapping binding regions are pulled down with a certain rate. For this study, we used used a pull-down rate of either 1.0 or 0.5, i.e., all or half of the overlapping fragments are used.
- Reverse transcription: For each fragment, one of the following modifications can be applied to the 5^′^-end read:
  - The read start is shifted to one of the simulated crosslink sites within the current binding region according to a given truncation probability (set to 0.7).
  - The read start is shifted to any other position within the fragment according to a given off-target truncation probability (set to 0.1).
- Size selection: To obtain a broad range of cDNA lengths, we keep reads with underlying fragment lengths between 30 and 140 nt (as recently recommended in [5]).

To evaluate the performance of our method, we developed a workflow to simulate realistic iCLIP-seq data, starting from aligned RNA-seq data and known binding regions. The workflow simulates the main steps of the iCLIP/eCLIP protocols (see Fig. 6), as follows:

- Fragmentation: To obtain RNA fragment lengths comparable to those of iCLIP experiments (30–300 bp, as described in [6]), we first simulate new fragment lengths using a normal distribution (mean: 165 bp, standard deviation: 50 bp).

In addition to the RBP binding signal, we also simulated background noise, which can be, for example, caused by sticky RNAs or by the binding of non-specific background proteins [10]. We did this by applying the steps described for the list of known common background binding regions published in [11], while varying pull-down rates, truncation probability, and the number of crosslink sites within a region. We supplement those regions with reads randomly sampled from RNA-seq data (1 *%*). Further details of the simulation used are described in Additional file 1: Section 2.

### PureCLIP hidden Markov model

**Fig. 7:**
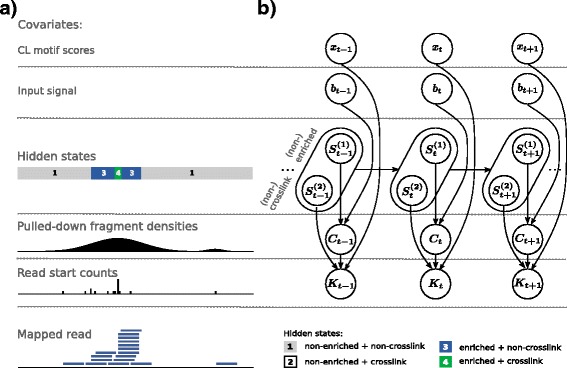
Summary of the hidden Markov model framework. **a** Starting from mapped reads (*bottom*), observations are deduced (individual read start counts, which are further smoothed to obtain pulled-down fragment densities) and combined with additional covariates (*top*) to reconstruct the most likely sequence of hidden states (*middle*). **b** Graphical representation of the corresponding non-homogeneous hidden Markov model. CL crosslink-associated

PureCLIP uses a HMM to infer crosslink sites from aligned single-nucleotide CLIP-seq data. At each position *t*, it utilizes two types of information (Fig. 7a): the pulled-down fragment density *C*_*t*_, which is used to infer whether the position is enriched or non-enriched in protein bound fragments, and the read start count *K*_*t*_, which is used to infer whether it is a crosslink or non-crosslink site. The four resulting hidden states are (1) *non-enriched* + *non-crosslink*, (2) *non-enriched* + *crosslink*, (3) *enriched* + *non-crosslink*, and (4) *enriched* + *crosslink*. For clarity, we separate them into two state variables. One represents the enrichment state:

1 $$ S^{(1)} = \left\{\begin{array}{ll} 0, & \text{if \textit{non-enriched},} \\ 1, & \text{if } {enriched}, \end{array}\right.  $$

and one represents the crosslink state:

2 $$ S^{(2)} = \left\{\begin{array}{ll} 0, & \text{if \textit{non-crosslink},} \\ 1, & \text{if } {crosslink}. \end{array}\right.  $$

Our goal is to identify positions that are *enriched* + *crosslinked* (see state (4) in Fig. 7a). Transitions between all four states are allowed and their probabilities are assumed to be homogeneous over the transcriptome. These transition probabilities are computed with the Baum–Welch algorithm [32], as the expected number of transitions based on computed posterior probabilities (for further details, see Additional file 1: Section 3). For each state, we model a distinct emission probability distribution, which is described in the next paragraph.

#### Joint emission probabilities and inference

We exploit the hierarchical structure of the two observed signals, i.e., the pulled-down fragment density (*C*_*t*_) and the count of read starts (*K*_*t*_), to specify the model. First, we model the fragment density *C*_*t*_, for both the *non-enriched* and the *enriched* states. The fragment densities are non-negative continuous values with a right-skewed distribution, which can be approximately described by a gamma distribution (see Additional file 1: Figures S2–S5). Furthermore, we do not want to fit the model to the large proportion of sites that have a very low density or no read start to improve both the efficiency and the robustness of the model (for details and the effect on the performance of PureCLIP, see Additional file 1: Section 3.1). Accordingly, we use a left-truncated gamma distribution (LTG), which is fitted only to sites with at least one read start:

3 $$\begin{array}{*{20}l} & P\left(C_{t} = c_{t} \mid S^{(1)} = s_{1}\right) = f_{\text{LTG}}(c_{t} ; \mu_{s_{1}}, \lambda_{s_{1}}, tp), \\  & s_{1} \in \{0,1\}, \end{array} $$

where $\mu _{s_{1}}$ and $\lambda _{s_{1}}$ denote the mean and the shape parameter of the distribution and *tp* is the truncation point. The corresponding probability density function is

4 $$\begin{array}{*{20}l} f_{\text{LTG}}&\left(c_{t}; \mu_{s_{1}}, \lambda_{s_{1}}, tp\right) =  \\ & \frac{1}{1- \frac{\gamma \left(\lambda_{s_{1}}, \frac{\lambda_{s_{1}} \cdot tp}{\mu_{s_{1}}}\right)}{\Gamma\left(\lambda_{s_{1}}\right)}} \cdot \frac{c_{t}^{\lambda_{s_{1}}-1} \exp \left({-\frac{\lambda_{s_{1}} \cdot c_{t}}{\mu_{s_{1}}}} \right)}{\left(\frac{\lambda_{s_{1}}}{\mu_{s_{1}}}\right)^{\lambda_{s_{1}}} \Gamma \left(\lambda_{s_{1}}\right)} \\ & \text{for}\ c_{t} > tp\ \text{and } \mu_{s_{1}}, \lambda_{s_{1}} > 0,  \end{array} $$

where $\gamma (\lambda _{s_{1}}, {\lambda _{s_{1}} \cdot tp}/{\mu _{s_{1}}})$ denotes the lower incomplete gamma function and $\Gamma (\lambda _{s_{1}})$ the ordinary gamma function. The parameters $\mu _{s_{1}}$ and $\lambda _{s_{1}}$ need to be learned, while *tp* is fixed (see Additional file 1: Section 3.1.2 for details).

When looking at the read start counts *K*_*t*_, we expect an increased count at crosslink sites due to underlying truncation events. Therefore, we model the read start counts *K*_*t*_ for both the *non-crosslink* and the *crosslink* states. For state *s*_2_, the probability of observing *k*_*t*_ read starts is computed given the number of trials *n*_*t*_ and the probability $p_{s_{2}}$ for each read to start at position *t*. To be precise, *n*_*t*_ is the number of fragments/trials from which a certain fraction results in reads starting at position *t*. In this case, it is unknown. However, we can use a surrogate value $\hat {n}_{t}$ directly deduced from the position’s pulled-down fragment density *c*_*t*_ by a simple rescaling (for details, see Additional file 1: Section 3.2.1 and Figure S7). We model the emission probability distribution with a zero-truncated binomial distribution (ZTB):

5 $$\begin{array}{*{20}l} &P\left(k_{t} | c_{t}, S^{(2)}_{t} = s_{2}\right) = f_{\text{ZTB}}\left(k_{t}; \hat{n}_{t}, p_{s_{2}}\right),\\  & s_{2} \in \{0,1\}. \end{array} $$

The probability density function is

6 $$\begin{array}{*{20}l} &f_{\text{ZTB}}\left(k_{t}; \hat{n}_{t}, p_{s_{2}}\right) =  \\ &\left\{\begin{array}{ll} 0, & \quad \text{if~} k_{t} = 0, \\ \frac{1}{1 - (1-p_{s_{2}})^{\hat{n}_{t}}} \binom{\hat{n}_{t}}{k_{t}} p_{s_{2}}^{\hat{n}_{t}} \left(1 - p_{s_{2}} \right)^{\hat{n}_{t} - k_{t}}, & \quad \text{if~} k_{t} \geq 1. \\ \end{array}\right. \end{array} $$

The probability parameters *p*_0_ and *p*_1_ need to be learned. *p*_1_ reflects a protein-specific truncation rate at crosslink sites. A zero-truncated binomial distribution is preferred here as we do not want to fit the distributions to the large number of sites with no read starting (for further details, see Additional file 1: Section 3.2.2).

Given the described emission probability distributions, we compute the probability of a joint observation. Note that *C*_*t*_ and *K*_*t*_ are not conditionally independent, but since $\hat {n}_{t}$ is directly deduced from *c*_*t*_, the emission probability for the joint observation can be factorized accordingly (see Fig. 7b for a graphical summary):

7 $$\begin{array}{*{20}l} P&\left(c_{t}, k_{t} | S^{(1)}_{t} = s_{1}, S^{(2)}_{t} = s_{2}\right) = \\  &P\left(c_{t}| S^{(1)}_{t} = s_{1}\right) \cdot P\left(k_{t} | c_{t}, S^{(2)}_{t} = s_{2}\right) =\\  &f_{\text{LTG}}\left(c_{t} ; \mu_{s_{1}}, \lambda_{s_{1}}, tp\right) \cdot f_{\text{ZTB}}\left(k_{t}; \hat{n}_{t}, p_{s_{2}}\right). \end{array} $$

Finally, we use posterior decoding to determine the most likely hidden state for each position, and with that, all *enriched* + *crosslink* sites (*s*_1_=*s*_2_=1). Each such called crosslink site has an associated score, namely the log posterior probability ratio of the first and second most likely state:

8 $$ \operatorname{score}_{t} = \log \left(\frac{P(\text{1st likely state} | Y_{1:T})}{P(\text{2nd likely state} | Y_{1:T})} \right),  $$

where *Y*_1:*T*_ denotes the observed data for all positions. In a second step, the called crosslink sites can be further combined to binding regions based on their distance.

We use the Baum–Welch algorithm [33] to learn the parameters of the HMM, i.e., the transition probabilities and the parameters of the four emission probability distributions (see Additional file 1: Section 3 for details of the implementation). Moreover, to reduce the computational costs, we trained the HMM on a subset of the chromosomes (Chr1–Chr3 for pooled data and Chr1–Chr6 for individual replicates). This had no impact on the quality of the estimates.

#### Estimation of the pulled-down fragment density

To model the fragment density, we cannot use positions-wise read counts, since they will be strongly influenced by truncation events in the neighborhood. Instead, we smooth the read start counts *k* to estimate the density of pulled-down fragments at each position. This is done using a kernel density estimation [18] with a Gaussian kernel function *K*. The latter assigns a higher weight to nearby read starts, while still considering read starts that are further away, thereby providing a better estimate for the underlying pulled-down fragment density. We compute the smoothed signal at position *t* using

9 $$  c_{t} = \frac{1}{h} \sum_{i = t-4h}^{t+4h} k_{i} \cdot K \left(\frac{t-i}{h} \right),  $$

where *h* is the kernel bandwidth. Positions within four bandwidths are considered.

### PureCLIP non-homogeneous HMM

We aim to correct for different sources of biases that influence the observed signals within iCLIP/eCLIP data. Accordingly, we incorporate position-wise external data as covariates into the HMM using generalized linear models to obtain non-homogeneous emission probabilities. Besides this, we currently assume that transitions probabilities between the four states do not change along the transcriptome. Numerical optimization techniques are then used in the Baum–Welch algorithm to find the emission probability parameters that maximize the conditional expectation of the data.

#### Incorporation of a non-specific background signal

Without additional information, we assume that for each enrichment state *s*_1_, the fragment density *c*_*t*_ follows a left-truncated gamma distribution:

10 $$ c_{t} | S^{(1)}_{t} = s_{1} \sim \operatorname{LTG}\left(\mu_{s_{1}}, \lambda_{s_{1}}, tp\right).  $$

If a non-specific background signal is given, e.g., from an input control experiment, PureCLIP incorporates this as position-wise covariates into the model. This is done using a (left-truncated) gamma generalized linear model. The objective is to learn the correlation between the covariate *b* and the mean parameter $\mu _{s_{1}}$ of each enrichment state *s*_1_. The underlying multiplicative effect of the background signal *b*_*t*_ on the expected mean $\mu _{s_{1},t}$ is modeled using a log link function:

11 $$ \log \left(\mu_{s_{1},t}\right) = \alpha_{s_{1},0} + \alpha_{s_{1},1} b_{t}.  $$

Note that we assume each enrichment state *s*_1_ to have a constant shape $\lambda _{s_{1}}$ across the entire range of covariate values.

A numerical optimization is performed in the Baum–Welch algorithm to learn the parameters $\alpha _{s_{1},0}$, $\alpha _{s_{1},1}$, and $\lambda _{s_{1}}$ (see Additional file 1: Section 4.1). In this study, we used the log fragment density of the input experiments as covariates, computed using a kernel density estimation with the same bandwidth as used for target fragment density, i.e., 50 bp.

#### Incorporation of CL motif scores

Without additional given information, the read start counts *k*_*t*_ are modeled using a zero-truncated binomial distribution:

12 $$ k_{t} | S^{(2)}_{t} = s_{2} \sim \operatorname{ZTB}\left(\hat{n}_{t}, p_{s_{2}}\right),  $$

for each enrichment state *s*_2_. If we assume that we have learned *m* enriched CL motifs from the input data (described in the next section), then we can compute for each position *t* and motif *i*∈1,…,*m* a corresponding motif match score *x*_*i*,*t*_≥0, containing information about the position’s crosslinking affinity. PureCLIP uses a logistic regression for each crosslinking state *s*_2_ to model the expected binomial probability parameter $p_{s_{2}}$ based on the position-wise CL motif score *x*_*i*,*t*_:

13 $$\begin{array}{*{20}l} \ln \frac{p_{s_{2},t}}{1-p_{s_{2},t}} &= \beta_{s_{2},0} + \beta_{s_{2},i} x_{i,t}, \\ i &= \arg\max_{j \in 1,\dots,m} x_{j,t}, \quad x_{j,t} \geq 0. \end{array} $$

Since the majority of positions have no CL motif match, i.e., a CL motif score of 0, we compute $\beta _{s_{2},0}$ using these sites as was done in the basic PureCLIP model. Further, since we assume that each site matches only one CL motif (i.e., the motif with the highest score is chosen), the parameters $\beta _{s_{2},1}, \dots, \beta _{s_{2},m}$ are learned independently of each other using Brent’s method (see Additional file 1: Section 4.2).

#### Computation of CL motif scores

The computation of position-wise CL motif scores, which can be used as covariates by PureCLIP, is done in a preprocessing step:

1. We call crosslink sites on the input data using the basic version of PureCLIP.
2. We run DREME (meme suite v4.11.3) [22] with the parameters -norc -k 6 -4 on 10-bp windows spanning the called input crosslink sites, while using 10-bp windows 20 bp upstream and downstream as the control (DREME uses Fisher’s exact test).
3. We use FIMO (meme suite v4.11.3) [23] with the parameters (–thresh 0.01 –norc) to compute occurrences of those motifs within the genome and their corresponding scores. If one position overlaps multiple CL motif occurrences, the one with the highest score is chosen.

### Implementation

PureCLIP is a command-line tool implemented in C++ using SeqAn [34], the GNU Scientific Library [35], and Boost [36]. OpenMP [37] is used for parallelization.

### Comparison with previous crosslink site detection strategies

We compared PureCLIP with the following methods: *simple threshold*, CITS [16], Piranha [13], and CLIPper [19]. *Simple threshold* and CITS detect crosslink sites at single-nucleotide resolution and therefore, can be directly compared with PureCLIP.

Piranha and CLIPper are strand-specific peak-calling methods and cannot be directly compared to PureCLIP; therefore, their performance was assessed in combination with CITS. In detail, we take the intersection between peaks reported by Piranha (*p* value threshold 0.001) or CLIPper (default threshold) with CITS crosslink sites (default *p* value threshold) and score the resulting sites in two different ways: according either to the peak caller (referred to as Piranha ^sc^ or CLIPper ^sc^) or to CITS (referred to as CITS ^sc^).

The scores assigned were used to assess the performance of the strategy for different sensitivity thresholds. Further details of the method’s application and the parameter choice are described in Additional file 1: Section 5.

### Evaluation on real data based on bona fide binding regions

To assess the performance of the different strategies in detecting target-specific crosslink sites for the PUM2 and RBFOX2 datasets, we used the sequence motifs that were described for each of those proteins (Additional file 1: Figure S1). FIMO [23] (–thresh 0.001 –norc) was used to compute genome-wide motif occurrences. Next, for each called crosslink site, the distance to the closest motif start site was identified. The precision was defined as the percentage of all called sites that are located within 2 bp of a motif occurrence (Fig. 3).

For the protein U2AF2, its known predominant binding site ∼11 nt upstream of 3^′^ splice sites was used for evaluation. Ensembl release 75 annotations were used to compute the distance of each called crosslink site to the closest 3^′^ splice site. The precision is then defined as the percentage of all called sites that are located 11±4 nt upstream of a 3^′^ splice site.

### Computation of bias-corrected replicate agreement

For the evaluation based on the replicate agreement, only sites with calls at the exact same nucleotide position in both replicates were considered as agreeing. In all comparisons, the replicate dataset with the larger library size was chosen as a reference for the evaluation and this is referred to as replicate 2 in the following. We report for each given number of called sites *x* in replicate 1 (corresponding to a certain sensitivity threshold), the percentage that were also called within the top *x* ranking sites in replicate 2.

To compute the bias-corrected replicate agreement, we count only sites that additionally (1) have sufficient enrichment over the input signal and (2) are not contained in common background regions [11] or in CL motifs (for PUM2 and RBFOX2).

To determine the sites whose pulled-down fragment densities are enriched over the input, we chose an individual threshold for each protein dataset based on its distribution of log-fold enrichment (for details, see Additional file 1: Section 7). CL motif occurrences were obtained with FIMO as described previously, while using all matches with a score >0. Common background binding regions were taken from [11], using only regions observed in at least six different CLIP-seq datasets, and extending them upstream and downstream by 200 bp.

## Additional file

Additional file 1 Additional figures and more detailed information about the computational methods and the results. (PDF 1352 kb)
