# Supplementary material for: PureCLIP: capturing target-specific protein–RNA interaction footprints from single-nucleotide CLIP-seq data
Source: Genome Biol. 2017 Dec 28;18:240. doi: 10.1186/s13059-017-1364-2 (PMC5746957; doi:10.1186/s13059-017-1364-2)
Supplement: Additional file 1 — Additional figures and more detailed information about the computational methods and the results. (PDF 1352 kb) [file 13059_2017_1364_MOESM1_ESM.pdf]

PureCLIP: capturing target-specific protein-RNA interaction footprints from single-nucleotide CLIP-seq data

Supplementary material

Sabrina Krakau [1*], Hugues Richard [2†], and Annalisa Marsico[1,3†]

[1]Max Planck Institute for Molecular Genetics, 14195 Berlin, Germany
[2]Sorbonne Universités, UPMC Univ Paris 06, CNRS, IBPS, UMR 7238, Laboratoire de Biologie Computationnelle et Quantitative (LCQB), 75006 Paris, France
[3]Freie Universität Berlin, 14195 Berlin, Germany
[*]Correspondence: krakau@molgen.mpg.de
[†] Equal contribution

Contents

1 Data **2**

2 Simulation of iCLIP/eCLIP-seq data **2**

3 PureCLIPs hidden Markov Model **3**
3.1 Emission probabilities: 'Non-enriched' and 'enriched' states . . . 3
3.1.1 Left-truncated gamma distribution . . . 3
3.1.2 Parameter estimation . . . 4
3.2 Emission probabilities: 'Non-crosslink' and 'crosslink' states . . . 9
3.2.1 Estimation of n_t . . . 9
3.2.2 Parameter estimation . . . 9
3.3 Initialization . . . 14

4 PureCLIPs non-homogeneous hidden Markov Model **14**
4.1 Incorporation of non-specific background signal . . . 14
4.2 Incorporation of CL-motifs . . . 15
4.2.1 Learned CL-motifs . . . 15
4.2.2 Incorporation of CL-motif scores . . . 16

5 Comparison against previous strategies **16**

6 Dependence of PureCLIPs performance on the bandwidth parameter **19**

7 Replicate agreement **21**

8 Evaluation at the level of binding regions **25**

1 Data

Protein	iCLIP or eCLIP	Binding characteristics	Cell line	Accession no.	Total reads	Filtered reads	Input
PUM2	eCLIP	sequence motif	K562	GSE91965	28 648 140	2 211 125	yes
RBFOX2	eCLIP	sequence motif	K562	GSE92030	38 506 096	11 178 409	yes
U2AF2	eCLIP	upstream of 3' splice site	K562	GSE92143	25 644 172	8 679 323	yes
U2AF2	iCLIP	upstream of 3' splice site	HeLa	E-MTAB-1371	22 905 120	12 060 930	no

Table S1: Summary of the datasets used in this study. All experiments have two replicates, whose reads were pooled prior to the actual analysis (except when accessing the replicate agreement), but after PCR duplicate removal.

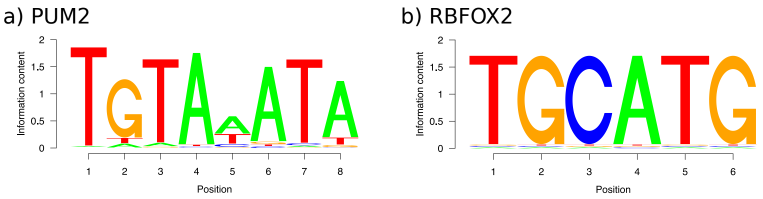

Figure S1: Sequence motifs used for PUM2 and RBFOX2 to define *bona fide* binding regions, retrieved from the ATtRACT database `https://attract.cnic.es`.

2 Simulation of iCLIP/eCLIP-seq data

The simulation for this study is based on the total RNA-seq dataset ENCSR000CPY from ENCODE (cell line K562), aligned with STAR. In order to obtain a realistic distribution of target binding regions we use FIMO [4] (–max-stored-scores 800000 –thresh 0.01 –norc) to compute PUM2 motif occurrences within the genome. Only motif occurrences located within transcripts and a minimal distance of 100 bp to the next annotated splice site were used. Note that we start the simulation with the 5'-ends of the given RNA-seq fragments and adjust the 3'-ends accordingly to the simulated length (step (1), Fig. 6). The read length is set to 50 bp initially, and is shortened accordingly, if individual RNA fragments become shorter during the simulation.

When simulating non-specific background noise, we use the list of known common background binding regions published in [6], and the following variations to the core workflow:

- We pull-down fragments overlapping such regions with a 'pull-down' rate between 0.0 and 1.0 proportionally to the original 'importance' scores for the background binding regions as reported in [6].
- The truncation probability is slightly decreased (0.6), assuming less specific crosslink sites within background binding regions.

- As background binding regions are typically longer than the used motif occurrences, we increased the number of simulated crosslink sites within a region: $c_i \in \{4, \ldots, 10\}$.

The aligned reads are then written to a BAM file, the simulated binding regions and crosslink sites to BED files. Note, that while the read lengths are adjusted according to simulated fragment ends and possible truncations, in the current version only dummy sequences are written to the BAM file, i.e. a realigning is not possible.

3 PureCLIPs hidden Markov Model

We constrain the HMM to *covered regions*. Given the bandwidth parameter h (in this study 50), a covered region is defined as a region that 1) contains read start sites with a distance of at most $4h$ bp between two successive sites, and 2) is extended by $2h$ bp beyond the outer read start sites. Reads with no other read starting within $4h$ bp, referred to as *singleton* reads, are discarded.

Transition and emission probabilities parameters of the HMM are estimated using the Baum-Welch algorithm, which is a special case of the expectation-maximization (EM) algorithm for discrete state space models. It iteratively alternates two steps: computing the posterior probabilities of hidden states given the observations, and then finding the parameters that maximise the conditional expectation of the data. We proceed in sequential Baum-Welch blocks to learn the parameters of the two types of observations, namely the parameters of left-truncated gamma (LTG) distributions modelling the observed fragment density c_t given the 'enrichment' state $S_t^{(1)}$, and the parameters of the zero-truncated binomial (ZTB) distributions modelling the number of read starts k_t given 'crosslink' states $S_t^{(2)}$ and c_t. First the LTG parameters are learned with fixed initial ZTB parameters. Then, the ZTB parameters are learned using the preliminary LTG parameters before another Baum-Welch block is applied for a final update of the LTG parameters. Transition parameters are updated in each Baum-Welch iteration. Within each block, the iterations are terminated when for all parameters the change is below a certain threshold. In case of the LTG, there is no closed form solution to the maximisation of the conditional expectation so we use numerical optimisation techniques (for details see Section 3.1.2).

3.1 Emission probabilities: 'Non-enriched' and 'enriched' states

3.1.1 Left-truncated gamma distribution

The gamma distribution is a popular and flexible choice to model non-negative continuous values with a right skewed distribution and thus we use it for the fragment density values. We investigated the properties of the observed fragment densities with skewness-kurtosis plots (originally proposed by Cullen and Frey [1], implemented in the *fitdistrplus* R package [2] which was used here). For this we used all sites which are used by PureCLIP for fitting the 'non-enriched' and 'enriched' emission probability distributions: sites with at least one read starting while discarding singleton reads. Although we do not know the empirical distributions corresponding to the assumed components of our model ('non-enriched', 'enriched'), the skewness-kurtosis plots indicate that both the total fragment densities within real data, as well as the fragment densities from simulated data can be best modeled with the properties of a gamma distribution, in comparison for example to a lognormal distribution (see Fig. S2, S3). Furthermore, when dividing the observed values for different ranges of associated input fragment densities, the gamma distribution remains the best fitting distribution with respect to the skewness-kurtosis properties (see Fig. S4). This shows that also for the non-homogeneous HMM the gamma GLM is likely a good suitable model. A comparison of the compound emission probability distributions fitted by PureCLIP (in basic mode) to the empirical fragment densities as well as the resulting

classifications are shown in Figure S5.

Fitting the gamma distributions only to sites with at least one read start reduces the computational costs and additionally improves PureCLIP's robustness when incorporating input signal (see Fig. S6, right panel). More precisely, it prevents the learning of the regression coefficients α from being impaired by a large number of sites that have a very low fragment density, as well as a lower correlation between the target and the input signal. The same holds when discarding singleton reads (see Fig. S6a, right panel).

Truncated versions of the gamma distribution tend to display increased instability in the estimation of the mean and shape parameters. In order to regularise the estimates, we constrain the shape parameter $\lambda_{s_1=0}$ of the 'non-enriched' state ($S_t^{(1)} = 0$) to be $\lambda_0 \leq 1$, which in practice constrains the emission probability distribution to an exponential shape.

According to the computation of the kernel density estimate c_t described in eq. (9) in Materials and Methods, we fix the truncation point as the value of one single read start at the current location:

$$tp = \frac{1}{h} \cdot K\left(\frac{0}{h}\right). \tag{1}$$

3.1.2 Parameter estimation

While the scale parameter for conventional gamma distributions can be estimated using a closed formula, there exists no such formula to compute the mean parameter μ for the truncated gamma distribution. Therefore in the Baum-Welch algorithm we numerically estimate the mean μ_{s_1} and the shape λ_{s_1} parameters that maximize the conditional expectation of the data:

$$\begin{aligned}(\hat{\mu}_{s_1}, \hat{\lambda}_{s_1}) = \arg\max_{\mu_{s_1}, \lambda_{s_1}} \sum_t^T P(S_t^{(1)} = s_1 | Y_{1:T}) \cdot \Bigg[& (\lambda_{s_1} - 1) \cdot \log(c_t) - \frac{\lambda_{s_1} \cdot c_t}{\mu_{s_1}} \\ & - \lambda_{s_1} \cdot \log\left(\frac{\mu_{s_1}}{\lambda_{s_1}}\right) - \log(\Gamma(\lambda_{s_1})) \\ & - \log\left(1 - \frac{\gamma\left(\lambda_{s_1}, \frac{\lambda_{s_1} \cdot tp}{\mu_{s_1}}\right)}{\Gamma(\lambda_{s_1})}\right) \Bigg].\end{aligned} \tag{2}$$

Different methods exist to solve such optimization problems. One class of methods requires first order derivatives in order to use the gradient to find the extremum. However, in our case the derivatives of the truncated gamma distribution with respect to λ_{s_1} become very complex. We thus resorted to the Nelder-Mead optimization method (also called downhill simplex method), and its implementation in the GSL [3]. The Nelder-Mead method only requires the values of the function itself and not of any derivatives.

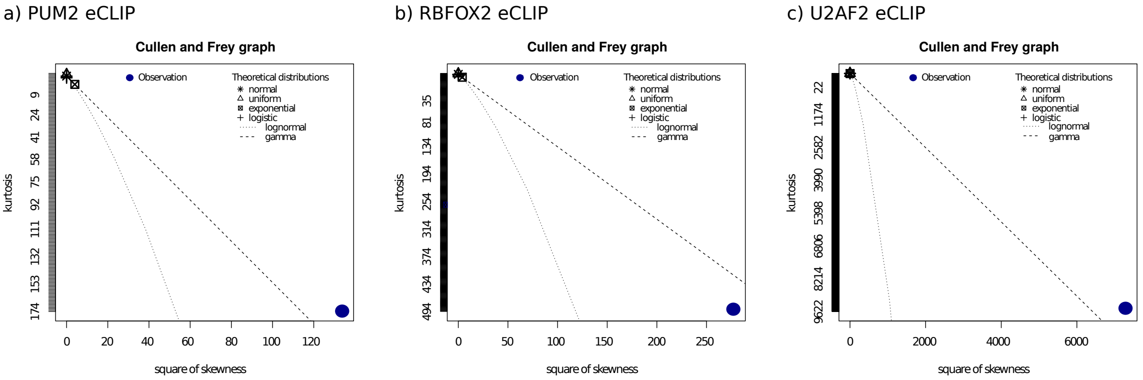

Figure S2: Skewness-kurtosis plot [1,2] for the pulled-down fragment densities. The fragment densities are taken from all sites (with $>= 1$ read starts, not including singleton reads) that are used by PureCLIP for fitting the 'non-enriched' and 'enriched' emission probability distributions.

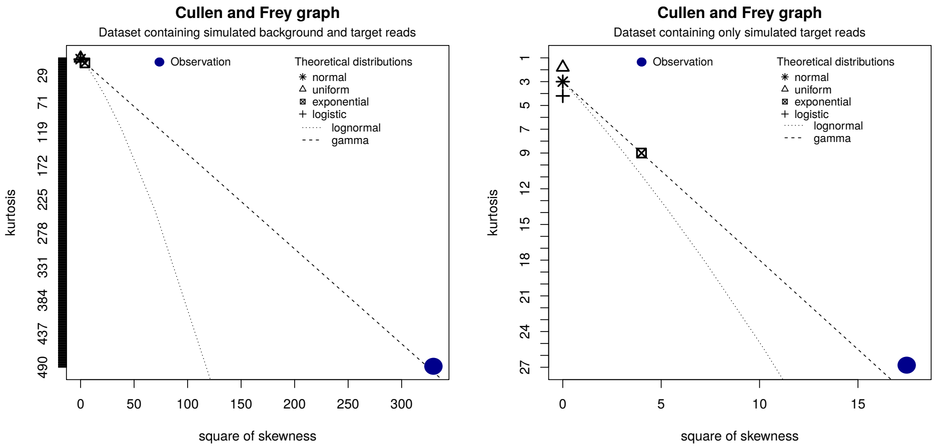

Figure S3: Skewness-kurtosis plot for fragment densities within simulated data. **Left:** Dataset containing mixture of simulated background and target signal. **Right:** Dataset containing only simulated target signal. The fragment densities are taken from all sites (with $>= 1$ read starts, not including singleton reads) that are used by PureCLIP for fitting the 'non-enriched' and 'enriched' emission probability distributions.

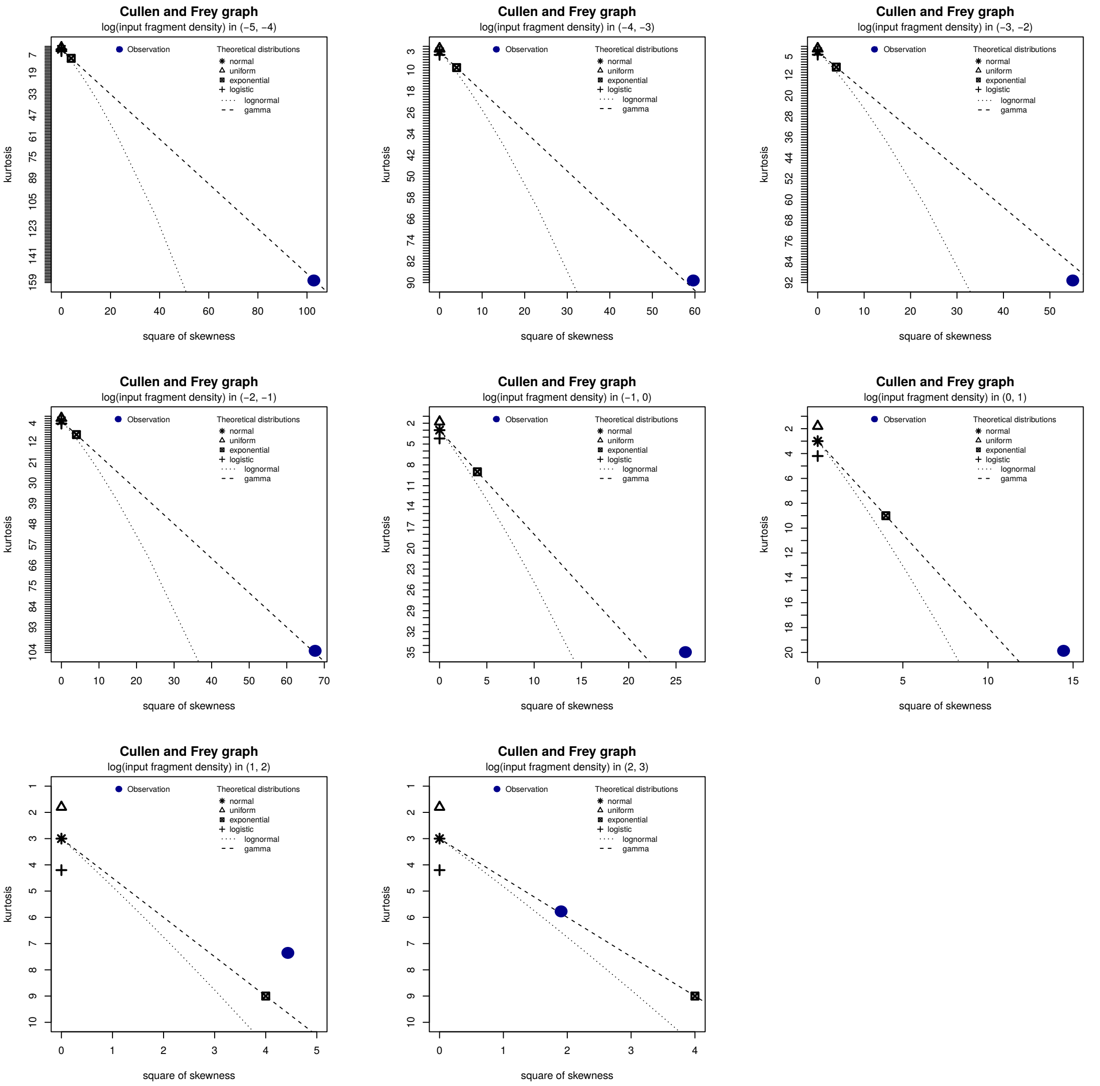

Figure S4: Skewness-kurtosis plot for PUM2 eCLIP fragment densities. Similar to Fig. S2a), but divided for different ranges of input fragment densities.

a) PUM2 eCLIP

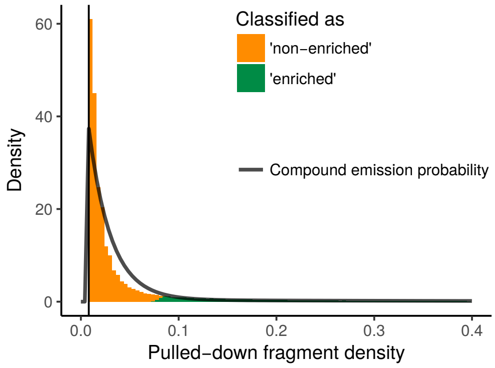

b) RBFOX2 eCLIP

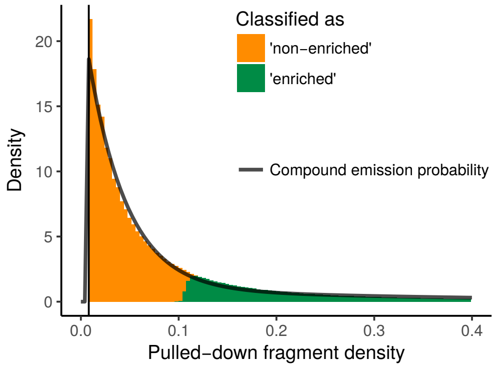

a) U2AF2 eCLIP

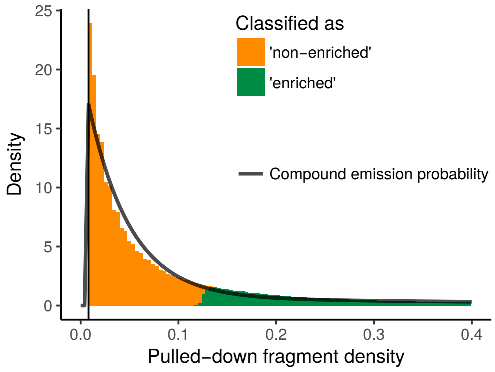

Figure S5: Distribution of empirical fragment densities (histogram) and the resulting classification by PureCLIP. The black line depicts the compound emission probability distribution of the fragment densities: $w_0 f_{LTG}(c_t; \mu_0, \lambda_0, tp) + w_1 f_{LTG}(c_t; \mu_1, \lambda_1, tp)$, were the weights w are derived from the corresponding posterior probabilities.

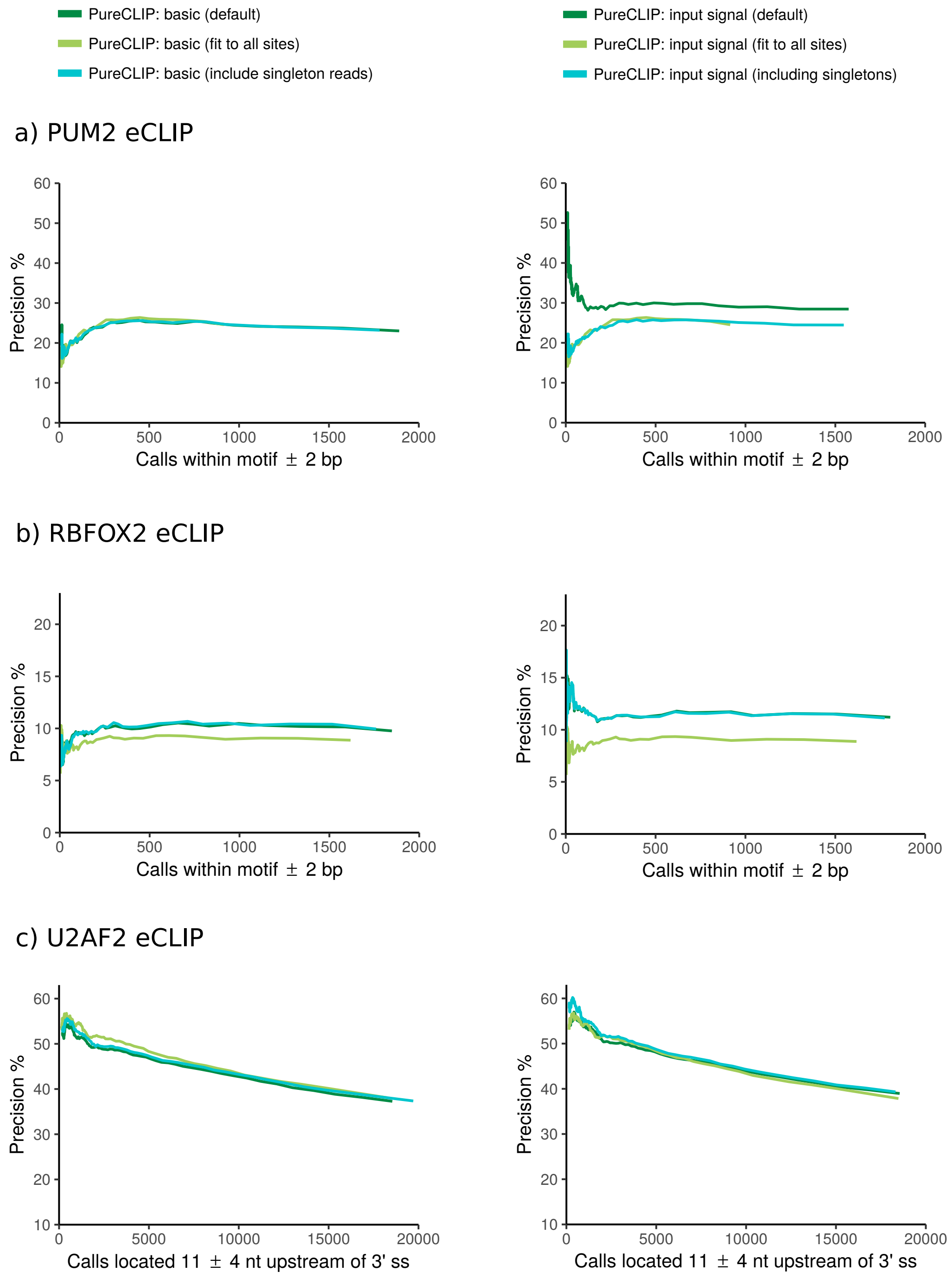

Figure S6: Precision of PureCLIP in default setting (using left-truncated gamma distributions fitted to sites with >= 1 read starts, discarding singleton reads) in comparison 1) to PureCLIP when using non-truncated gamma distributions fitted to all sites, i.e. also sites with no read starts, and 2) to PureCLIP when not discarding singleton reads. **Left panel:** PureCLIP in basic mode. **Right panel:** PureCLIP incorporating input signal (i.e. using gamma GLMs). Note that for 1) the computation is still constrained to covered regions.

3.2 Emission probabilities: ‘Non-crosslink’ and ‘crosslink’ states

3.2.1 *Estimation of* n_t

In order to model the read start counts using a binomial distribution, we need for each position the number of trials or fragments n_t from which a certain fraction results in read starts at this position (i.e. due the end of either a truncated or untruncated cDNA). For truncation based CLIP-seq data n_t is unknown and a reasonable estimate $\hat{n}_t$ is crucial in this context.

For this, we take advantage of the estimate c_t for the pulled-down fragment density and we expect a linear relationship between the observed read start counts n'_t within a spanning window and c_t, which we can model with linear regression:

$$n'_t = \lfloor \phi_0 + \phi_1 c_t + \varepsilon_t \rfloor. \tag{3}$$

We estimate the regression coefficients (ϕ_0, ϕ_1) using windows of size $2h$ (100 bp in this study) and use it afterwards to obtain $\hat{n}_t$ for each position (see Fig. S7).

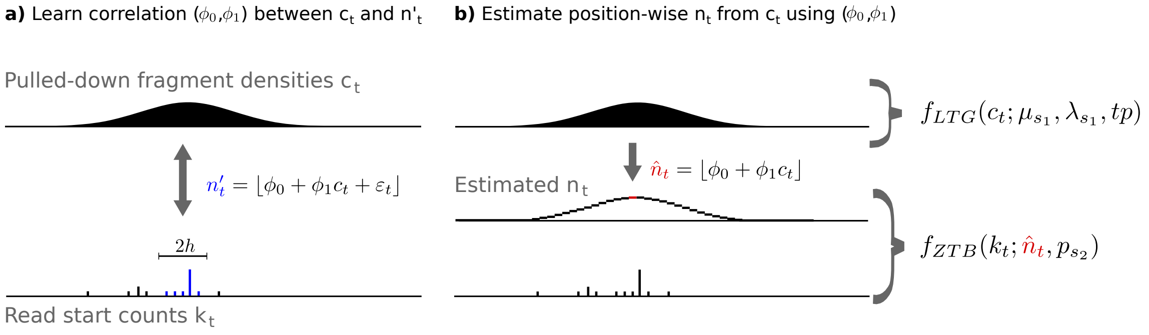

Figure S7: Estimation of n_t, i.e. the number of trials or fragments from which a certain fraction results in read starts at position t, for the zero-truncated binomial (ZTB) distribution: Instead of using the read start count within a spanning window of size $2h$, we estimate n_t based on the already computed position-wise fragment density c_t given the regression coefficients (ϕ_0, ϕ_1).

3.2.2 *Parameter estimation*

The (zero-truncated) binomial probability parameters p_0 and p_1, reflecting the read start probabilities corresponding to the ‘non-crosslink’ and ‘crosslink’ state, can be estimated with (see [7]):

$$\hat{p_s} = \sum_{t=1}^{T} \left(\frac{k_t - 1}{\hat{n}_t - 1} \right) P(S_t^{(1)} = 1, S_t^{(2)} = s_2 | Y_{1:T}) \qquad \text{for } \hat{n}_t \geq n_c. \tag{4}$$

To reduce the impact of background noise, we restrict this estimate by using the posterior probabilities for ‘enriched’ states as weights. Furthermore, since the largest fraction of read start counts occurs at positions with low $\hat{n}_t$ where the two distributions are not well distinguishable, we limit the parameter estimation for both p_0 and p_1 to positions with $\hat{n}_t \geq n_c$ a given threshold (default: $n_c = 10$). This prevents the algorithm from fitting the parameters mainly to these undistinguishable sites. As an example, Figures S8, S9 and S10 show empirical and learned distributions of read start counts for PUM2, RBFOX2 and U2AF2 eCLIP data, as well as the resulting classification.

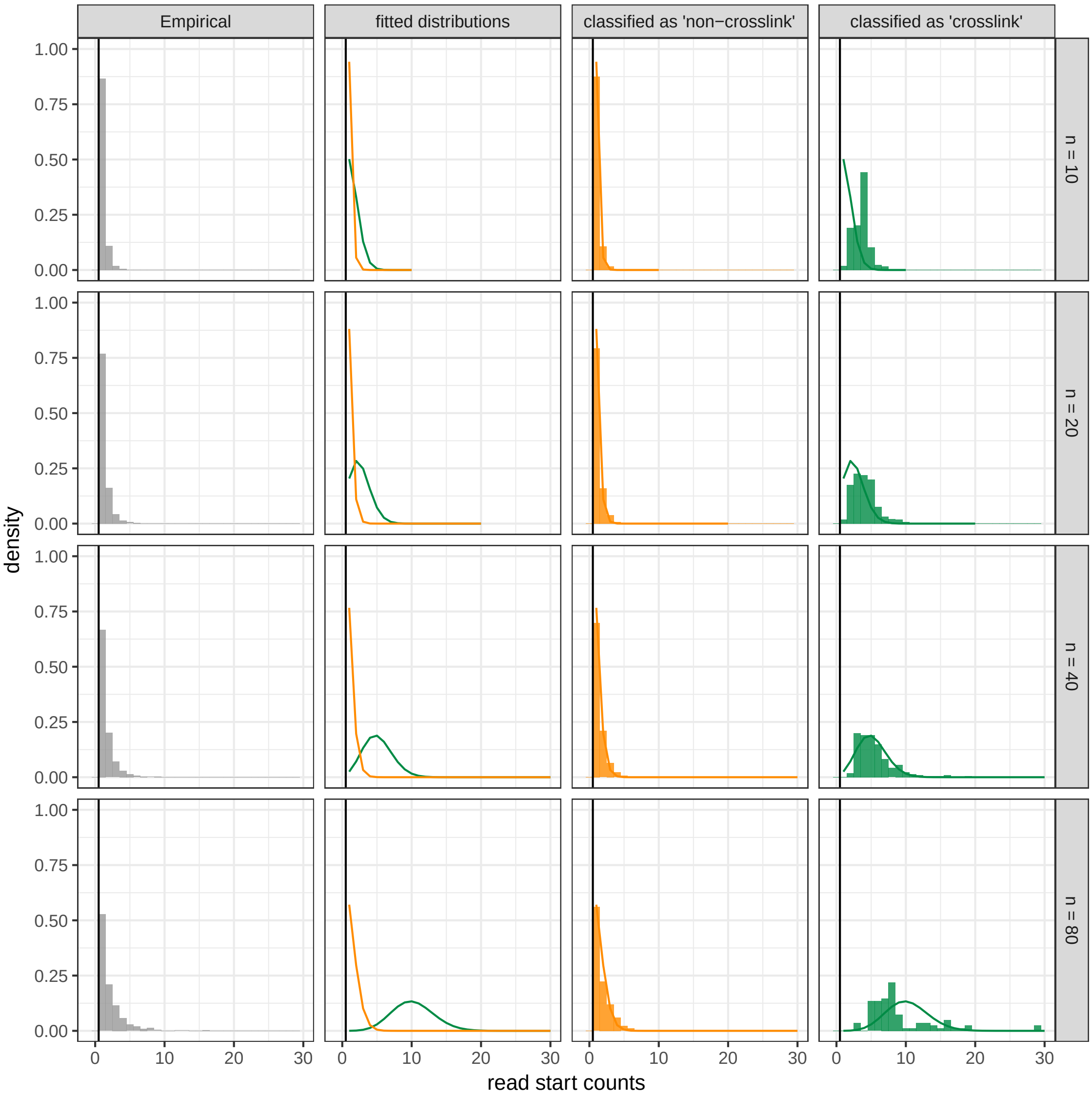

Figure S8: PUM2 eCLIP data: Read start count distributions for different values of $\hat{n}_t$ and fitted emission probability distributions. **Column 1:** Empirical distribution at sites that are used by PureCLIP for the parameter fitting (i.e. with $>= 1$ read starts). **Column 2:** Fitted emission probability distributions for the 'non-crosslink' (orange) and 'crosslink' (green) state. **Column 3:** Read start count distribution at sites classified as 'non-crosslink' (histogram), together with theoretical probability distribution (orange line). **Column 4:** Read start count distribution at sites classified as 'crosslink' (histogram), together with theoretical probability distribution (green line). The vertical black line represents the zero-truncation.

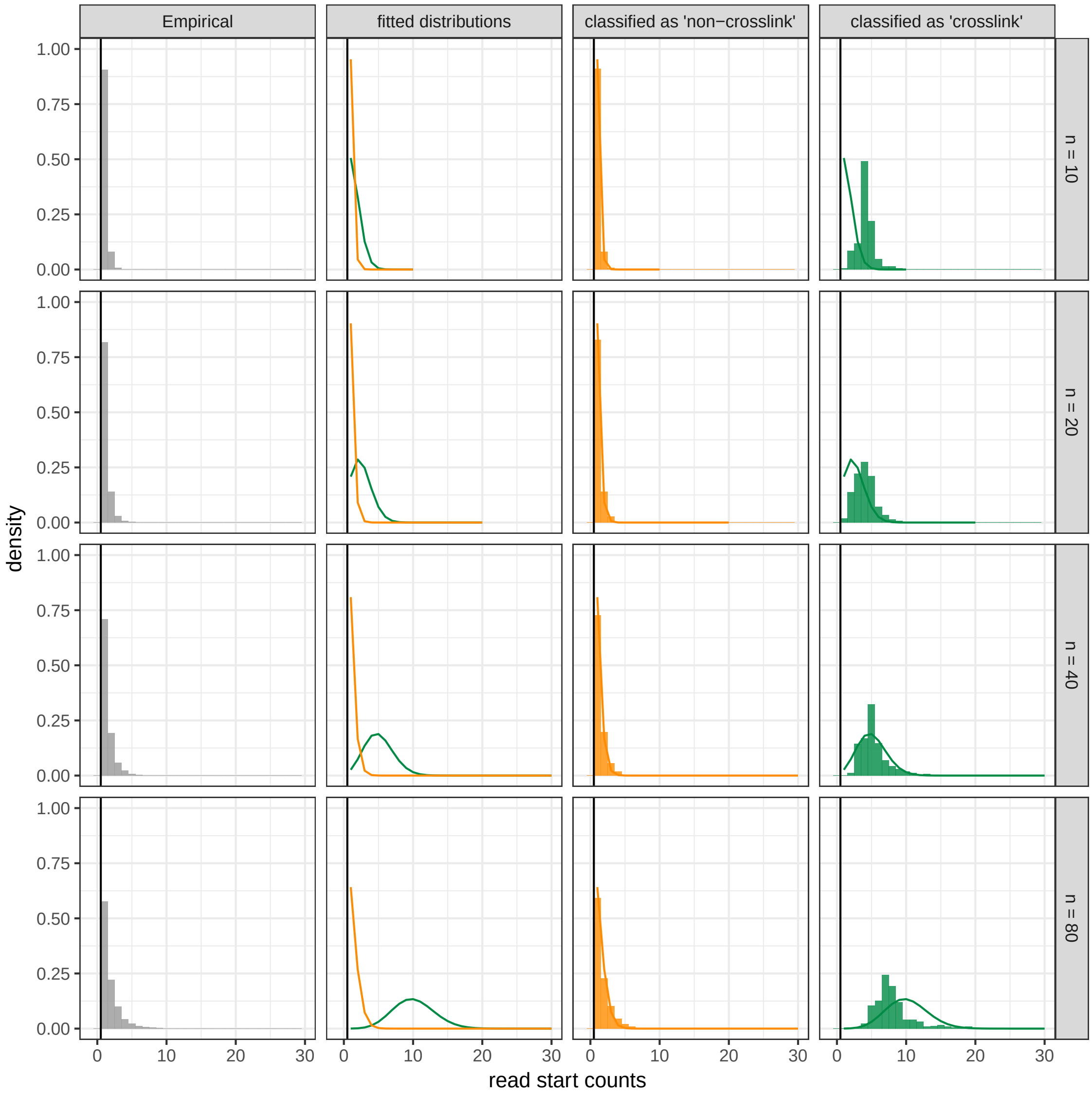

Figure S9: Same as Fig. S8, but for RBFOX2 eCLIP data.

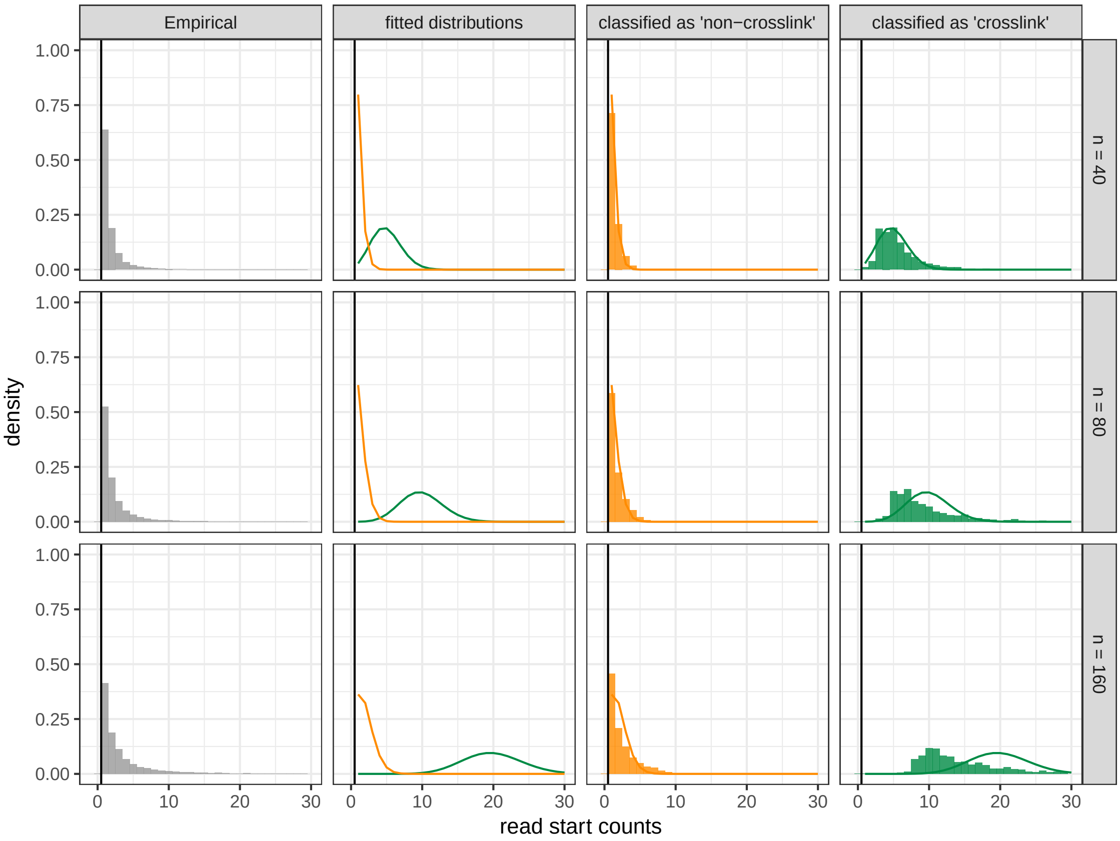

Figure S10: Same as Fig. S8, but for U2AF22 eCLIP data.

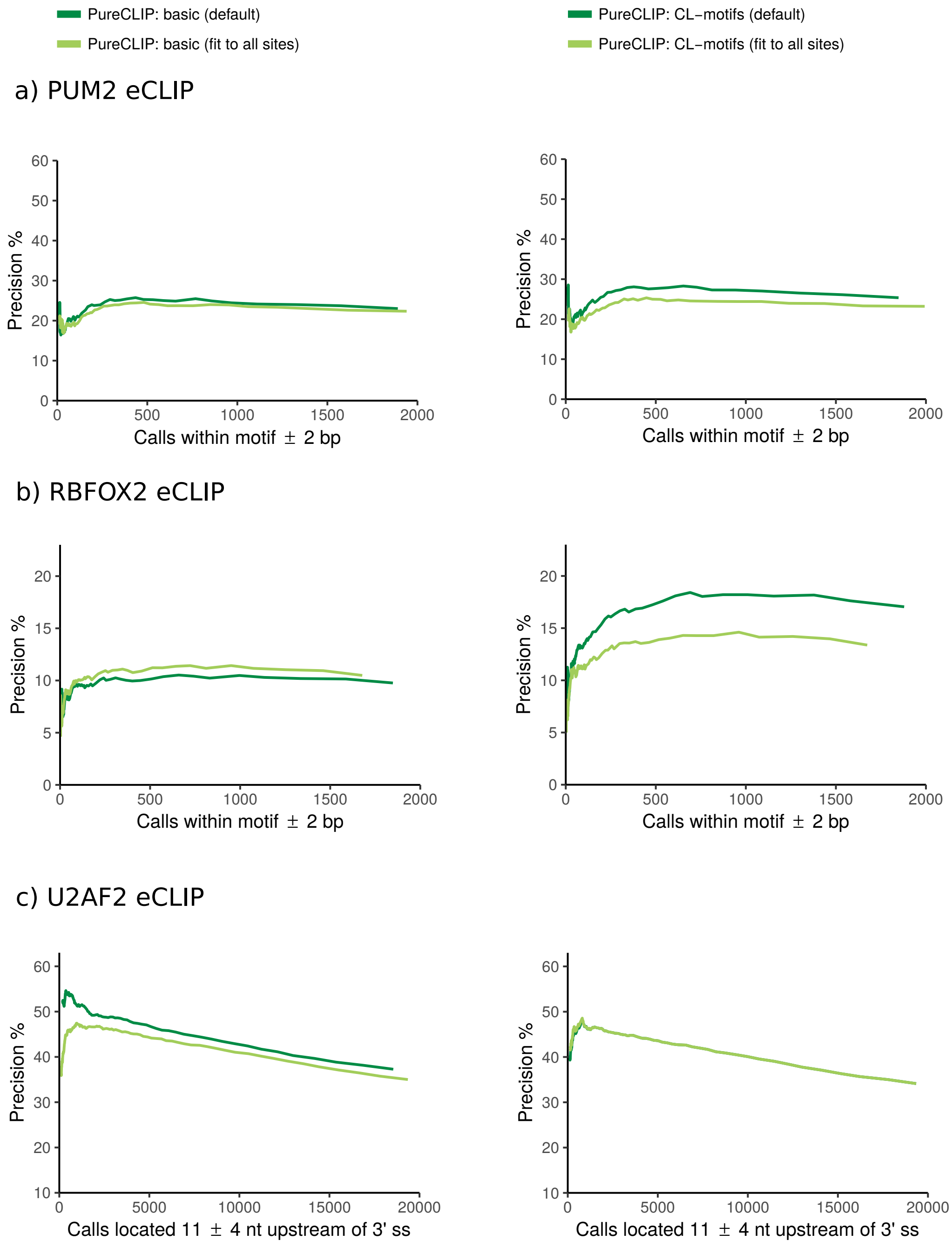

Figure S11: Precision of PureCLIP in default setting (using zero-truncated binomial distributions fitted to sites with >= 1 read starts) in comparison to PureCLIP when using non-truncated binomial distribution fitted to all sites, i.e. also sites with no read starts. **Left panel:** PureCLIP in basic mode. **Right panel:** PureCLIP incorporating CL-motifs. Note that in general the parameter fitting of the binomial is done only for the 'enriched' fraction (see Section 3.2.2).

3.3 Initialization

Because the Baum-Welch algorithm might fall into local optima with poor initializations, we set initial parameters within a preprocessing step. For the two LTG distributions, we start with a user defined discrete enrichment threshold e (default: 7), to compute a fragment density threshold c_e:

$$c_e = \frac{e}{h} \cdot K\left(\frac{0}{h}\right). \tag{5}$$

All sites with a fragment density $c_t >= c_e$ will be classified as 'enriched'. More precisely, we set the posterior probability of those sites to 0.999 for the 'enriched' state and to 0.001 for the 'non-enriched' state (and vice versa for all other sites). Next, we perform the M-step of the Baum-Welch algorithm. For the ZTB distributions, since only one parameter has to be estimated, their estimation is less dependent on the initialisation and thus we set it to default values ('non-crosslink' $p_0 = 0.01$; 'crosslink' $p_1 = 0.15$).

4 PureCLIPs non-homogeneous hidden Markov Model

4.1 Incorporation of non-specific background signal

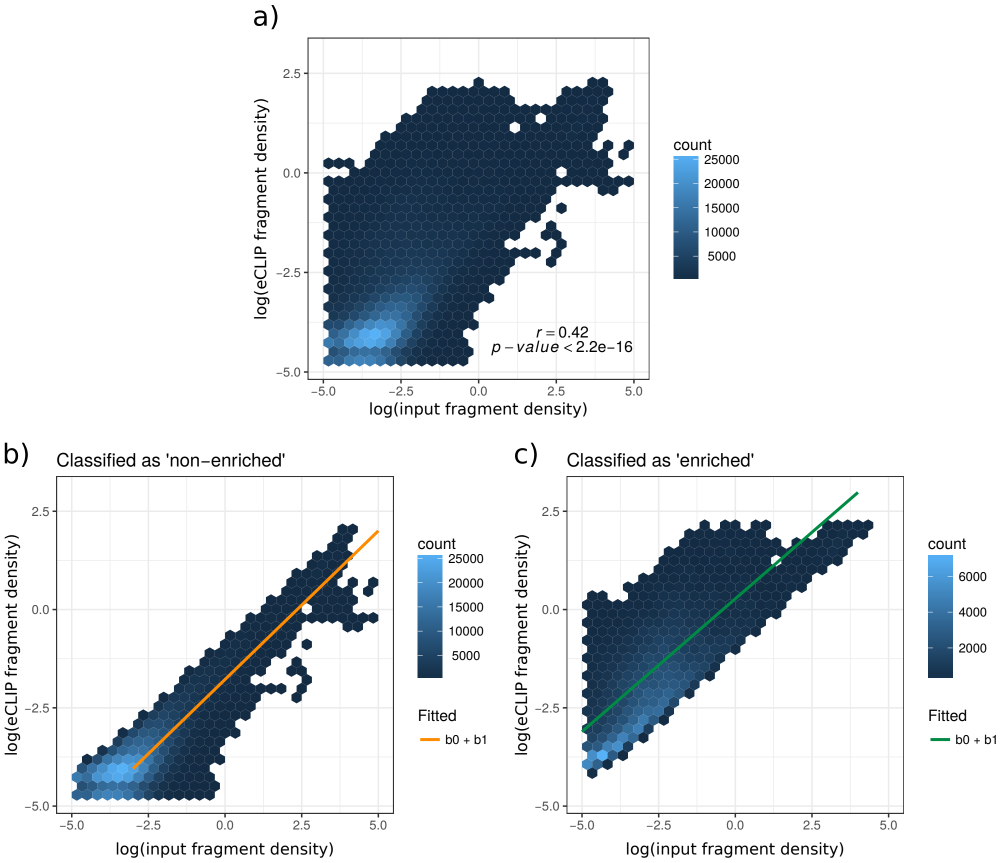

Figure S12: a) Correlation between PUM2 eCLIP pulled-down fragment density, and input fragment density at all positions with at least one read starting, not including singleton reads. b) same as a), but limited to the set of positions classified as 'non enriched' by PureCLIP (input signal covariate). c) same as a), but limited to the set of positions classified as 'enriched' by PureCLIP (input signal covariate). The expected mean pulled-down fragment densities for the 'non enriched' and 'enriched' states are represented as orange and green lines respectively.

We use a (left-truncated) gamma GLM in order to incorporate non-specific background signal as position-wise covariates b_t. Initial parameters are set with a slope of 0, i.e. $\mu_0 = \exp(\alpha_{0,0})$ and $\mu_1 = \exp(\alpha_{1,0})$. The mean μ_{s_1} and the shape parameters λ_{s_1} are then initialized using the same procedure as in the basic PureCLIP model.

The Baum-Welch algorithm is then used to maximize the conditional expectation of the data with respect to $\alpha_{s_1,0}$, $\alpha_{s_1,1}$ and λ_{s_1}, i.e. we aim to find:

$$\begin{aligned}(\alpha_{s_1,0}, \alpha_{s_1,1}, \lambda_{s_1}) = \arg \max_{\alpha_{s_1,0},\alpha_{s_1,1},\lambda_{s_1}} \sum_t^T \quad & P(S_t^{(1)} = s_1 | Y_{1:T}) \\ & \cdot \Bigg[(\lambda_{s_1} - 1) \cdot \log(c_t) - \frac{\lambda_{s_1} \cdot c_t}{\exp(\alpha_{s_1,0} + \alpha_{s_1,1} b_t)} \\ & + \lambda_{s_1} \cdot \log\left(\frac{\exp(\alpha_{s_1,0} + \alpha_{s_1,1} b_t)}{\lambda_{s_1}}\right) - \log(\Gamma(\lambda_{s_1})) \\ & - \log\left(1 - \frac{\gamma\left(\lambda_{s_1}, \frac{\lambda_{s_1} \cdot tp}{\exp(\alpha_{s_1,0}+\alpha_{s_1,1} b_t)}\right)}{\Gamma(\lambda_{s_1})}\right)\Bigg].\end{aligned} \tag{6}$$

Again the Nelder-Mead method is used for optimization. Importantly, in this context the shape parameter λ_0 of the 'non-enriched' state is not constrained to values smaller or equal to one. However, in order to the keep the parameter space limited, the 'non-enriched' shape parameter is ensured to be smaller or equal to the 'enriched' shape parameter.

4.2 Incorporation of CL-motifs

4.2.1 Learned CL-motifs

The analysis of the input data reveals similar polypyrimidine rich CL-motifs for the different datasets (see Fig. 4 and Fig. S13). It is worth noting, that the protein U2AF2 preferentially binds to poly(U) motifs, which means that for the U2AF2 eCLIP data the target motif and the top CL-motif largely coincide. On the one hand, this might lead to an efficient crosslinking at target-specific binding regions. On the other hand, this makes it more difficult for PureCLIP to distinguish target-specific crosslinks from non-specific crosslinks within CL-motifs. This is most likely the reason why the incorporation of CL-motifs do not lead to an improved precision for the analysis of this data (see Fig. 3).

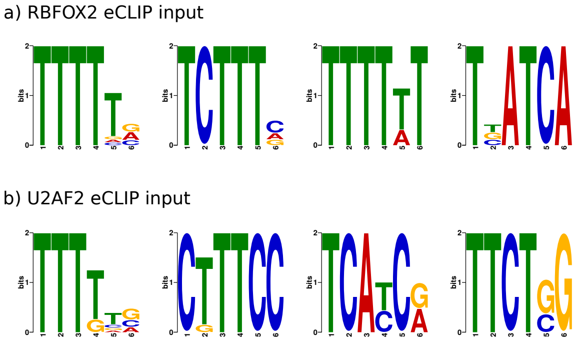

Figure S13: CL-motif analysis of RBFOX2 and U2AF2 eCLIP input data. Logo representation of the four top scoring motifs, computed as described in Materials and Methods.

4.2.2 Incorporation of CL-motif scores

When incorporating CL-motif scores into the model, the regression coefficients $\beta_{s_2,1}, \ldots, \beta_{s_2,m}$ for the m CL-motifs are learned independently from each other. Given $\beta_{s_2,0}$ and an indicator variable $I_{j,t}$ deduced from the position-wise motif match scores $x_{j,t}$, we aim to find for each $j \in 1, \ldots, m$ the parameter that maximizes the conditional expectation of the data :

$$\beta_{s_2,j} = \arg\max_{\beta_{s_2,j}} \sum_t^T P(S_t^{(1)} = 1, S_t^{(2)} = s_2 | Y_{1:T}) \cdot \Big[-\log\left(1 - (1 - p_{s_2,t})^{\hat{n}_t}\right) + k_t \cdot \log(p_{s_2,t}) + (\hat{n}_t - k_t) \cdot \log(1 - p_{s_2,t}) \Big] \cdot I_{j,t}, \quad (7)$$

where $I_{j,t}$ is defined as

$$I_{j,t} = \begin{cases} 1 & \text{if } x_{j,t} > 0 \ \& \ j = \arg\max_{i \in 1,\ldots,m} x_{i,t} \\ 0 & \text{else} \end{cases} \quad (8)$$

$$p_{s,t} = \frac{1}{1 + exp(-(\beta_{s,0} + \beta_{s,j} x_{j,t}))}.$$

The Brent's method is used within the Baum-Welch algorithm to find the maximizing parameters.

5 Comparison against previous strategies

CITS (v1.0.5) scripts were run with default parameters as described in the documentation (https://zhanglab.c2b2.columbia.edu/index.php/CTK_Documentation), except that we set '-gap 0' in order to turn of the merging of called crosslink sites within a distance of 25.

We run Piranha (v1.2.1) with a p-value threshold of -p 0.001 and a bin size -b of 20bp (as done in [11]). A drawback of Piranha is that when using BAM files as input, it calls peaks based on the left-most read positions, i.e. for reverse mapping reads based on read end sites instead of read start sites (see also [9]). To address this, we preprocessed the data and used BED files with position-wise read start counts as input for Piranha. In principle, Piranha can incorporate external data as bin-wise covariates. However, it would be up to the user to preprocess the data and choose an appropriate bin size.

We run CLIPper using the most recent master version at the time of preparing the manuscript (tagged as 'evaluation' at https://github.com/skrakau/clipper/ forked from https://github.com/YeoLab/clipper) and specified -s hg19. Since CLIPper uses annotations, it reports overlapping peaks in some cases. Therefore we choose the CLIPper peak with the lowest p-value when combining with CITS. It is worth noting that the eCLIP data published by the ENCODE consortium [8] is analysed using CLIPper followed by a post processing step, in which input control experiments are included to compute input normalized p-values [10]. Since the latter is not part of CLIPper, we do not include this post processing step in the comparison.

When comparing the performance of the different strategies for different sensitivity settings, the number of called sites (and the corresponding precision or replicate agreement) is given for each applied p-value or score threshold. We applied a large range of different thresholds corresponding to the given range of reported p-values or scores, however, the range of reported p-values or scores is limited for some methods. Thus the leftmost point of

each precision curve corresponds to the number of calls associated with the lowest p-value or the highest score that the corresponding strategy can report.

For the evaluations presented in this study we chose the parameters of the other methods as described. The results in Figure S14 show that PureCLIP also has a higher precision in detecting crosslink sites within PUM2 motif occurrences when using different p-value thresholds for the other strategies.

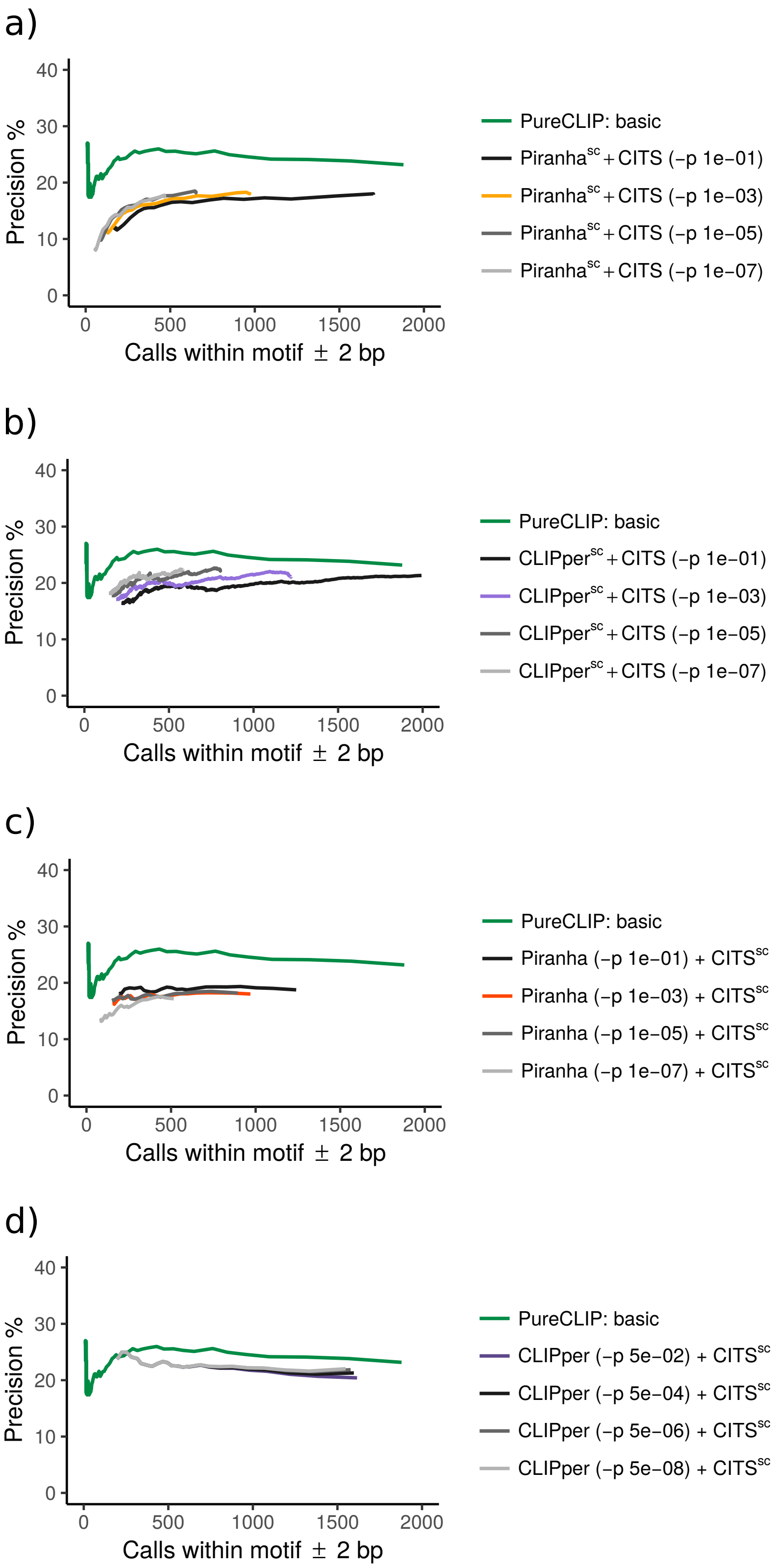

Figure S14: Influence of the applied p-value threshold for all the compared strategies on the precision in detecting crosslink sites within the *bona fide* binding regions (PUM2 eCLIP dataset).

6 Dependence of PureCLIPs performance on the bandwidth parameter

Figure S15 and S16 show the performance of PureCLIP using different bandwidth parameters in comparison to 'CLIPer + CITS^{sc}', which performs best among the previous strategies. While for different datasets different bandwidths would be optimal, the resulting differences in the precision are relatively minor compared to the difference to previous strategies. However, it is worth noting that the runtime and memory consumption of PureCLIP increases with increasing bandwidths. Due to this reason and since it produces a consistently good precision we chose a default bandwidth of 50 bp.

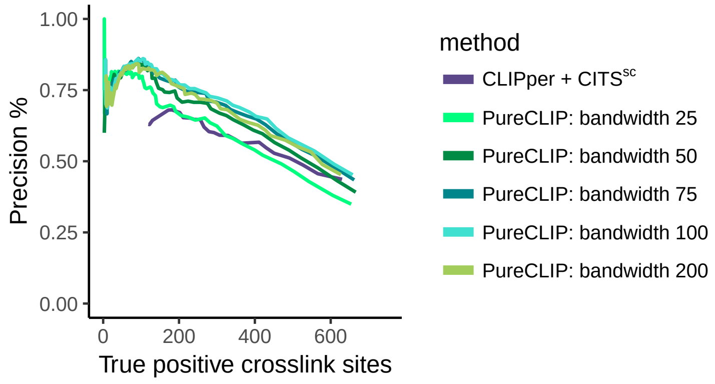

Figure S15: Robustness with respect to the bandwidth used for the estimation of the pulled-down fragment densities. The precision of PureCLIP (basic mode) when using different bandwidth parameters on simulated data (Set 3: 100% target pull-down rate, simulated background binding) is plotted at different sensitivity levels and compared to 'CLIPer + CITS^{sc}'.

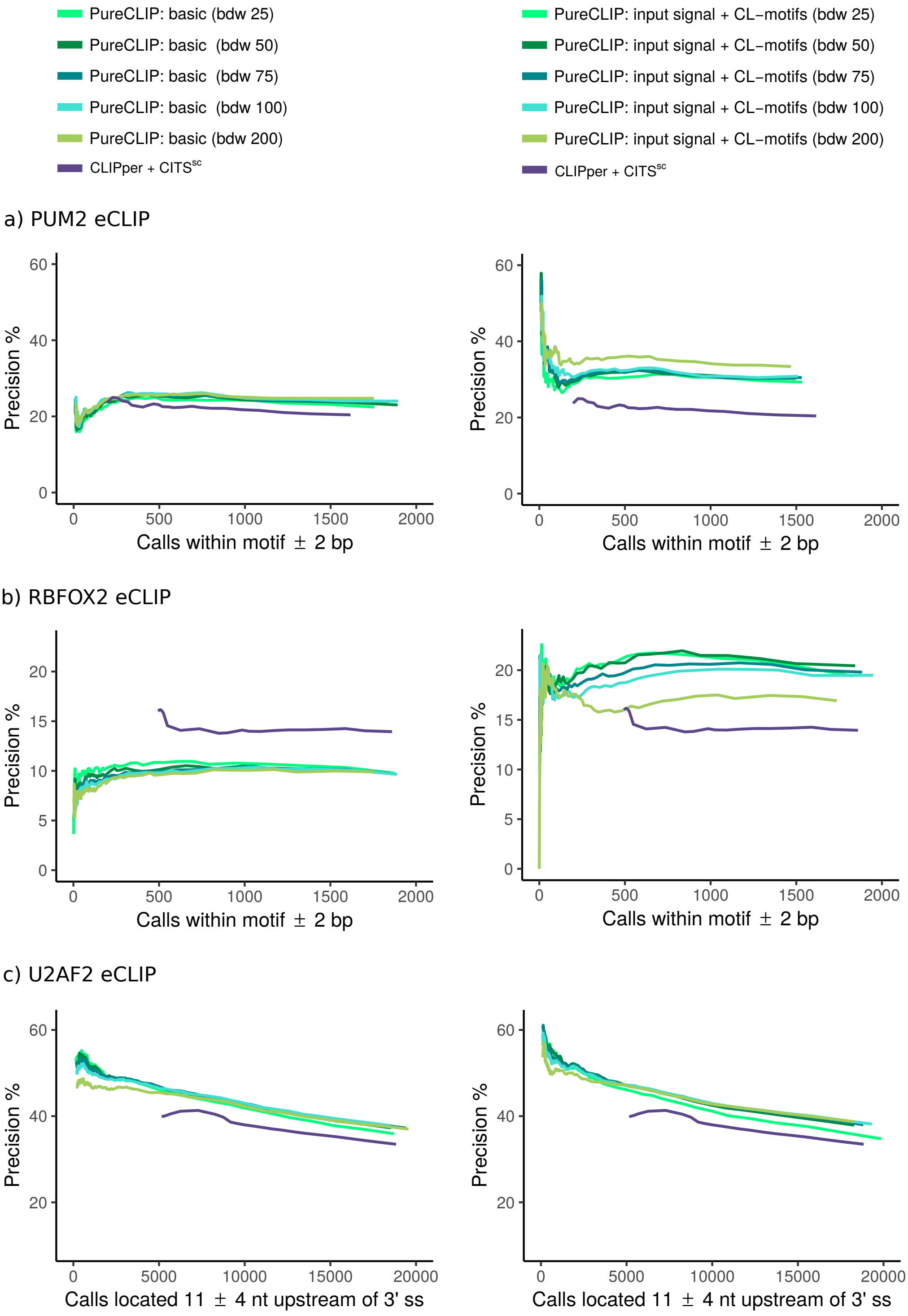

Figure S16: Robustness with respect to the bandwidth used for the estimation of the pulled-down fragment density. The precision of PureCLIP in detecting *bona fide* binding regions on real data when using different bandwidth parameters (bdw) is plotted at different sensitivity levels and compared to 'CLIPer + CITS^{sc}'. **Left panel:** PureCLIP in basic mode. **Right panel:** PureCLIP incorporating the input signal and CL-motifs as covariates. The same bandwidth is used to smooth the read start counts of the target and the input data.

7 Replicate agreement

For all strategies we explored the agreement of called crosslink sites between the replicates for PUM2, RBFOX2 and U2AF2 eCLIP data. Interestingly, when not accounting for any biases the *simple threshold* method achieves by far the highest agreement for all three eCLIP datasets (see Fig. S17a, S18a and S19a). However, we found that the *simple threshold* method also calls the most crosslinks at sites where the fragment density is not enriched over the input (see Fig. S17b, S18b and S19b). Furthermore, it calls more crosslinks at sites that are located within known background binding regions compared to other strategies (see Fig. S17e, S18e and S19e). These results demonstrate, that beside target-specific binding regions or crosslink sites, transcriptomic regions that are particularly prone to false positive calls contribute to the raw replicate agreement.

To determine the sites whose pulled-down fragment density is enriched over the input, we chose an individual threshold for each protein dataset. For this, we compare the log-fold enrichment of two distributions: the enrichments at *bona fide* crosslink sites and at all sites with at least one read starting. In this context, a *bona fide* site is a site that is called by PureCLIP and located within an occurrence of the protein's sequence motif. We then chose a threshold with the aim to separate the two distributions (see Fig. S17c, S18c and S19c).

Note that for U2AF2 the replicate agreement is not corrected for the sequence bias, since its target motif coincides with a common CL-motif. Thus, if we would compute the same bias corrected agreement as for the two other proteins, we would loose a large fraction of true positives. However, in comparison to other proteins, we also expect a higher crosslinking efficiency at target-specific interactions sites and, as a consequence, a less biased crosslink site detection (which would be in agreement to the relatively high achieved precision of all strategies in detecting crosslink sites within the known binding region $\sim$ 11 nt upstream of 3' splice sites, see Fig. 3).

The crosslink sites called by PureCLIP, in particular the top ranking sites, clearly show a higher bias corrected replicate agreement compared to previous strategies (see Fig. 5). Beside the described biases, there might be a remaining bias caused by different fragment densities. We expect that crosslink sites with a high fragment density are more likely to be called in both replicates. However, PureCLIP in general calls sites with lower fragment density compared to other strategies (see Fig. S17f, S18f and S19f). Thus, a potential bias due to fragment density would lead to an under-estimation of the replicate agreement for PureCLIP, compared to peak calling methods which benefit from calls in highly enriched regions

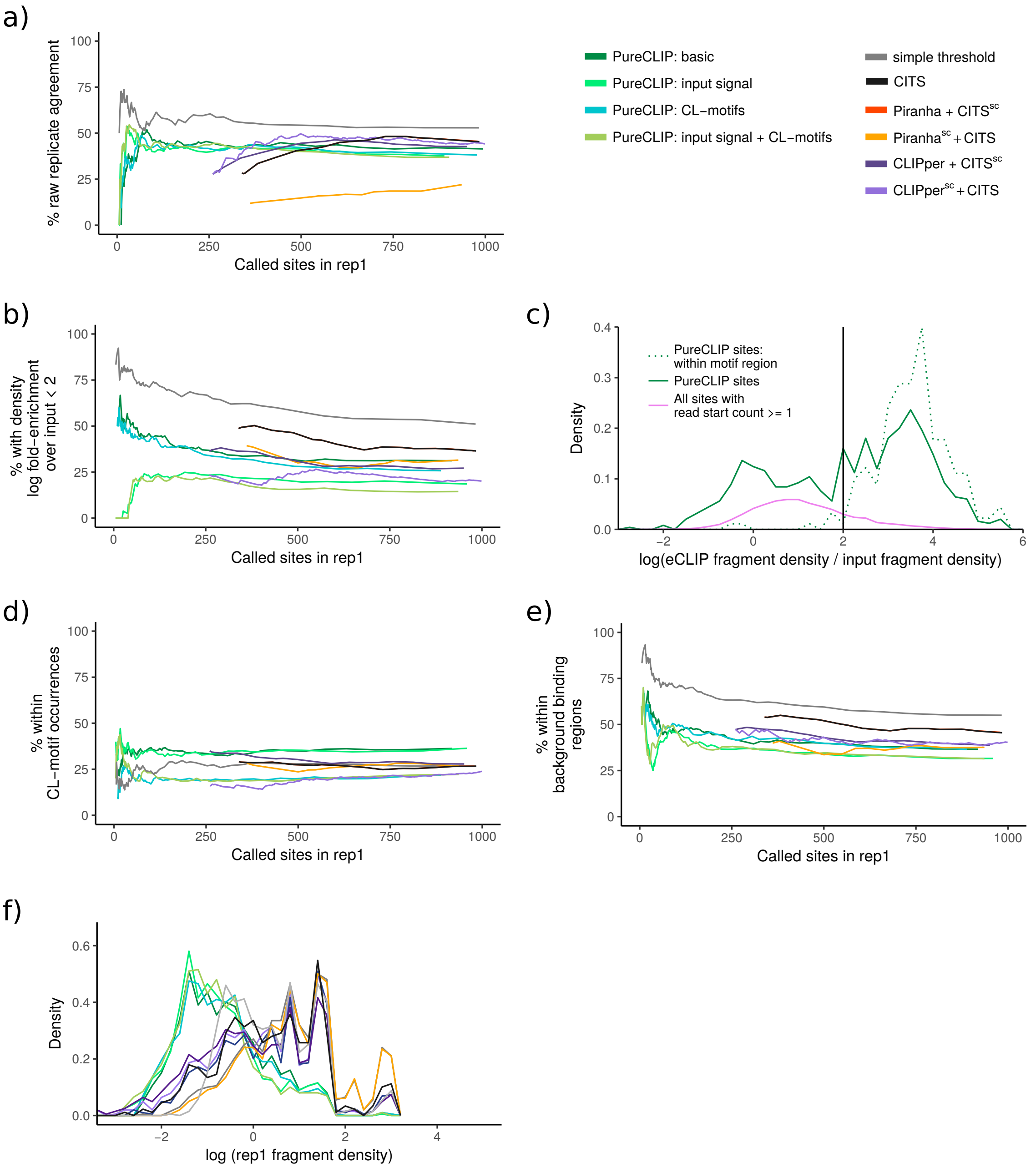

Figure S17: PUM2 eCLIP: replicate agreement and mapping of called crosslink sites within bias prone regions. **a,b,d,e)** For each given number x of called sites (corresponding to a certain sensitivity threshold) in replicate 1, we report the percentage that: **a)** were also found within the x top ranking called sites in replicate 2. **b)** have a log-fold enrichment over the input below 2 (i.e. bias related to highly abundant RNAs) **d)** are located within CL-motif occurrences (i.e. bias related to crosslinking sequence preferences) **e)** overlap with one of the background binding regions (see Material and Methods).
c) Distribution of log-fold fragment density enrichments over the input for sites called by PureCLIP (green), for sites called by PureCLIP and located within the PUM2 binding motif (dash green), and for all sites with at least one read start (pink). Based on the distribution at PureCLIP sites overlapping the known binding motif, a threshold of 2 is set for bias correction (black vertical line). **f)** Distribution of the log pulled-down fragment densities for the top 1000 ranking sites detected by each method.

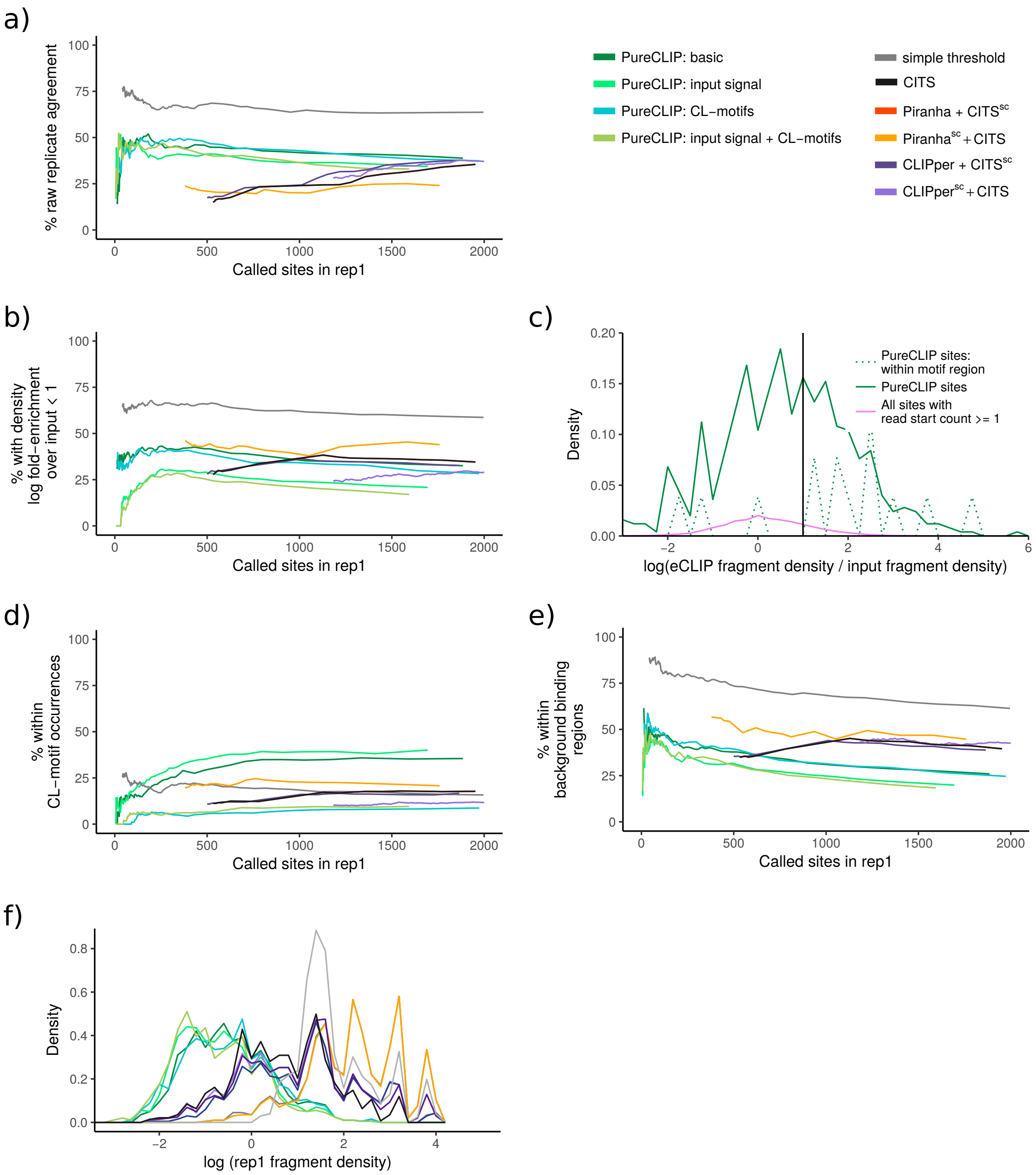

Figure S18: RBFOX2 eCLIP, bias correction of replicate agreement. Same as Figure S17, using a log-fold enrichment threshold of 1 (b, c).

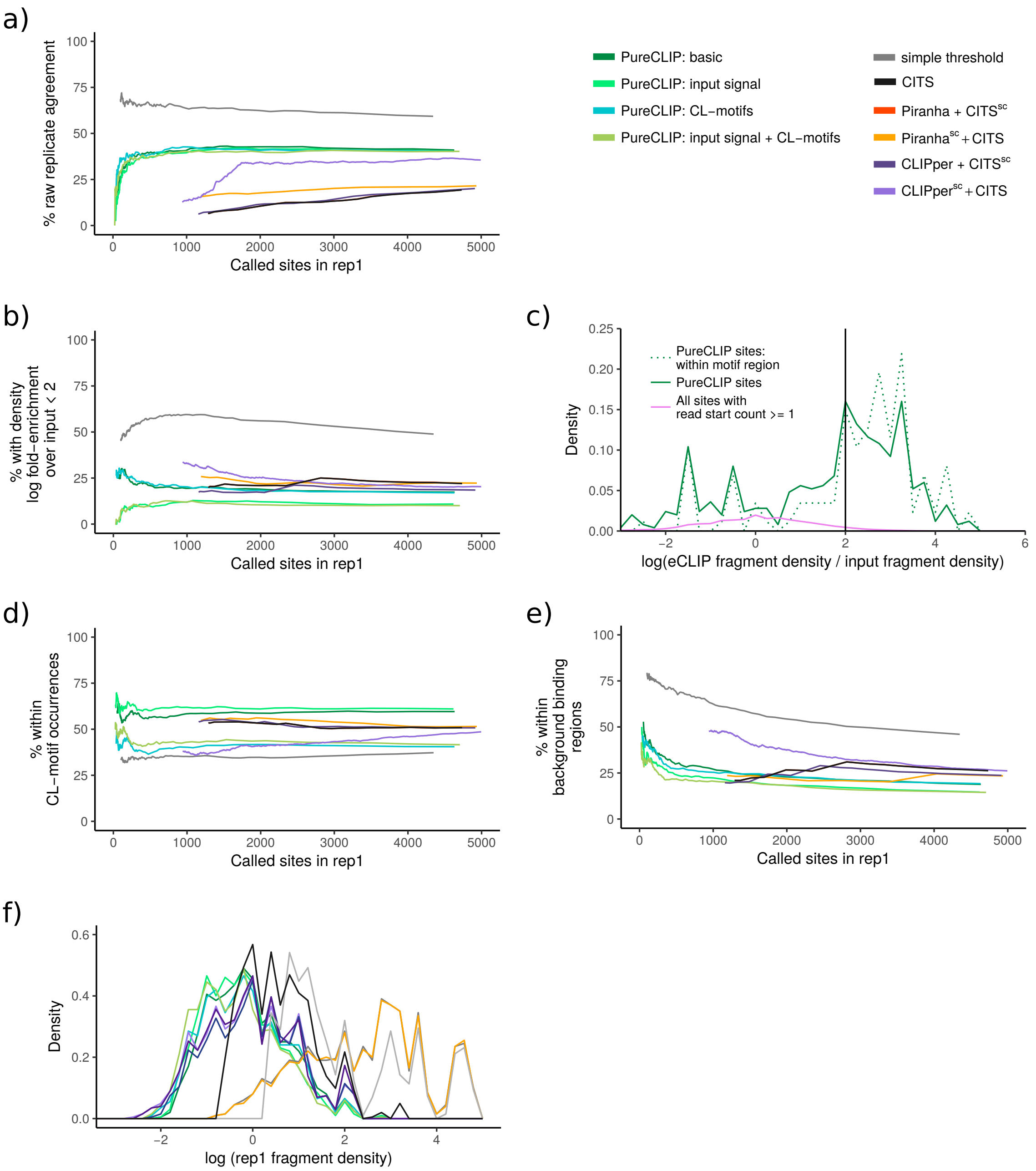

Figure S19: U2AF2 eCLIP, bias correction of replicate agreement. Same as Figure S17, using a log-fold enrichment threshold of 2 (b, c).

8 Evaluation at the level of binding regions

When investigating the performance of the different methods in detecting binding regions, we assume that for called regions a higher density of the target-specific motif corresponds to a higher accuracy. In order to estimate this accuracy we compute region-wise motif scores as follows:

- First we computed genome-wide motif occurrences together with a motif match score using FIMO [4] (–max-stored-scores 1600000 –thresh 0.1).
- Then we assigned these motif match scores to the center of the motif occurrences and applied a smoothing using a kernel density estimation (KDE_{motif}) [5] with a Gaussian kernel function.
- Each region gets scored by its mean KDE_{motif} value.
- A set of called binding regions is then evaluated based on the mean motif score.

The idea is to not only score the sites of a called region that match the motif, but also to score sites located close to the motif higher than sites further away. Large regions around a motif get lower scores than concise regions spanning the motif.
PureCLIPs performance for different sensitivity settings is then compared against the peak-calling methods CLIPper and Piranha. The results in Figure S20 show that PureCLIP in general reaches a higher accuracy in calling binding regions compared to the other evaluated methods. Importantly, PureCLIP reaches higher scores when using a smaller KDE_{motif} bandwidth of 5 bp instead of 10 bp (i.e. high scores located more narrowly around the actual motif occurrence), showing that it performs best in detecting concise regions around motifs (see Fig. S20, right panel).

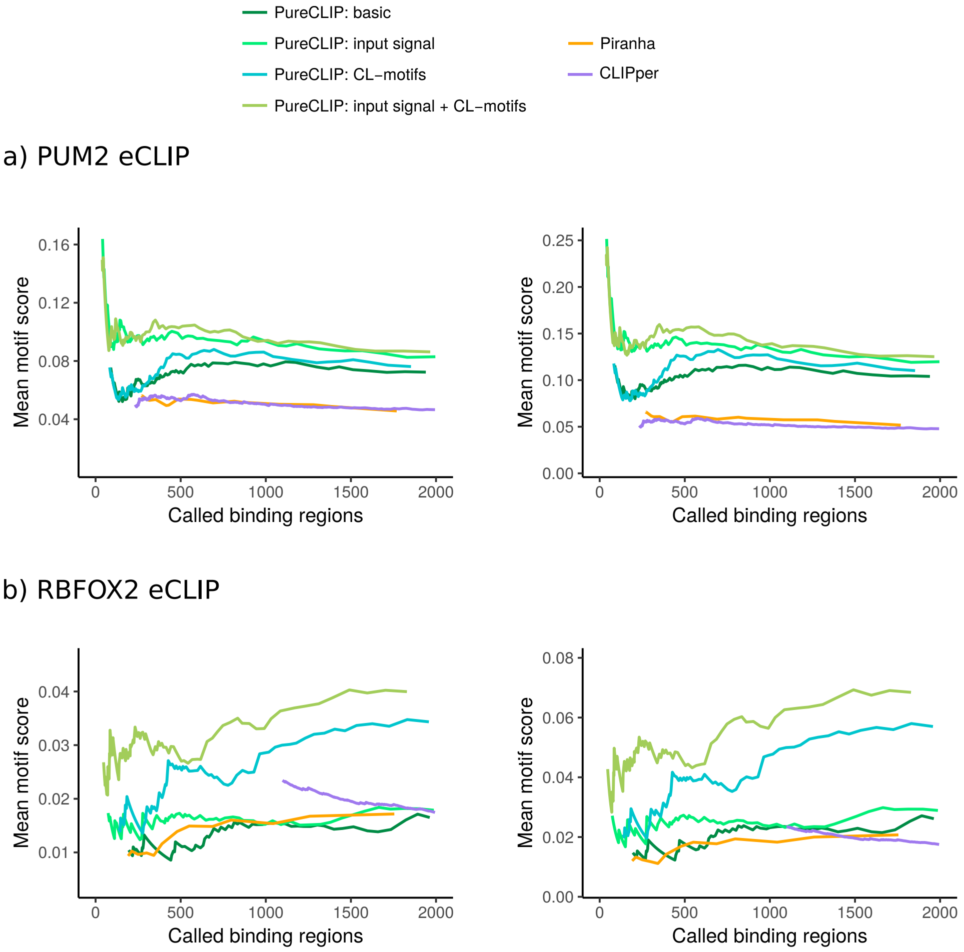

Figure S20: Comparison of PureCLIP's performance against existing peak-calling methods at the level of binding regions based on the motif density for the proteins PUM2 and RBFOX2. Motif scores are computed using a KDE$_{motif}$ bandwidth of 10 bp (left panel) and 5 bp (right panel).

References

[1] Alison Cullen and Cristopher Frey. Probabilistic Techniques in Exposure Assessment. In *Plenum Press: New York*. 1999.

[2] Marie Laure Delignette-Muller and Christophe Dutang. fitdistrplus : An R Package for Fitting Distributions. *Journal of Statistical Software*, 2015.

[3] M Galassi, J Davies, J Theiler, B Gough, G Jungman, P Alken, M Booth, F Rossi, and R Ulerich. GNU Scientific Library Reference Manual , ISBN 0954612078. *Library available online at http://www. gnu. org/software/gsl*, 2015.

[4] Charles E Grant, Timothy L Bailey, and William Stafford Noble. FIMO: scanning for occurrences of a given motif. *Bioinformatics*, 27(7):1017–1018, 2011.

[5] Emanuel Parzen. On estimation of a probability density function and mode. *The annals of mathematical statistics*, 33(3):1065–1076, 1962.

[6] P H Reyes-Herrera, C A Speck-Hernandez, C A Sierra, and S Herrera. BackCLIP: a tool to identify common background presence in PAR-CLIP datasets. *Bioinformatics*, page btv442, 2015.

[7] Paul R Rider. Truncated binomial and negative binomial distributions. *Journal of the American Statistical Association*, 50(271):877–883, 1955.

[8] Cricket A Sloan, Esther T Chan, Jean M Davidson, Venkat S Malladi, J Seth Strattan, Benjamin C Hitz, Idan Gabdank, Aditi K Narayanan, Marcus Ho, Brian T Lee, and Others. ENCODE data at the ENCODE portal. *Nucleic acids research*, 44(D1):D726–D732, 2016.

[9] Michael Uhl, Torsten Houwaart, Gianluca Corrado, Patrick R Wright, and Rolf Backofen. Computational analysis of CLIP-seq data. *Methods*, 2017.

[10] Eric L Van Nostrand, Gabriel A Pratt, Alexander A Shishkin, Chelsea Gelboin-Burkhart, Mark Y Fang, Balaji Sundararaman, Steven M Blue, Thai B Nguyen, Christine Surka, Keri Elkins, and Others. Robust transcriptome-wide discovery of RNA-binding protein binding sites with enhanced CLIP (eCLIP). *Nature methods*, 13(6):508–514, 2016.

[11] Yu-Cheng T Yang, Chao Di, Boqin Hu, Meifeng Zhou, Yifang Liu, Nanxi Song, Yang Li, Jumpei Umetsu, and Zhi John Lu. CLIPdb: a CLIP-seq database for protein-RNA interactions. *BMC genomics*, 16(1):51, 2015.
